# MetaRanker: precise profiling of antibiotic resistome risk in metagenomes by integrating abundance and genetic co-occurrence

**DOI:** 10.1128/aem.02422-25

**Published:** 2026-02-18

**Authors:** Zhenyu Guo, Yao Xiao, Junqiao Zhao, Zizhen Tang, Yufei Lin, Kun Yang

**Affiliations:** 1 Department of Pharmaceutical and Biological Engineering, School of Chemical Engineering, Sichuan University 617183, Chengdu, China; 2 Key Laboratory of Bio-Resources and Eco-Environment of Ministry of Education, College of Life Sciences, Sichuan University 615932 , Chengdu, China; Centers for Disease Control and Prevention, Atlanta, Georgia, USA

**Keywords:** antibiotic resistance gene, resistome, risk assessment, metagenomic analysis, gene quantification

## Abstract

The proliferation of antibiotic resistance genes (ARGs) in environmental microbiomes represents a major and growing threat to public health, creating a critical demand for precise and efficient tools to monitor resistance risk. Current approaches often depend on contig-based quantification or lack comprehensive risk indices, which compromises their accuracy and utility. To address this, we developed MetaRanker (https://github.com/SteamedFish6/MetaRanker), a computational pipeline that assesses resistome risk by integrating the abundance of ARGs, mobile genetic elements (MGEs), and virulence factors (VFs)—calculated directly from sequencing reads—with their genetic co-occurrence on contigs into a unified risk index (RI). This index reflects the potential for horizontal transfer and pathogen emergence. Evaluated using *in silico* and diverse real-world metagenomes (*n* = 353), MetaRanker demonstrated superior accuracy and stronger discriminatory power than existing methods. Its optimized compact database (29.6 MB) and alignment strategy reduced runtime by over 50% in comparison to MetaCompare 2.0 under identical hardware configurations (32 CPU cores, 128 GB RAM). Practical applications confirmed that MetaRanker effectively discriminates risk levels across environments (e.g., hospital wastewater versus natural soil) and quantifies risk mitigation through wastewater treatment. As a robust, lightweight, and sequencing-platform-agnostic tool, MetaRanker offers a powerful solution for comprehensive environmental resistome surveillance and evidence-based risk management.

**IMPORTANCE:**

The environmental reservoir of antibiotic resistance is a key contributor to the global health crisis of antimicrobial resistance. Effective surveillance and risk assessment of complex microbial communities are essential for prioritizing interventions and safeguarding public health. However, existing methods often provide fragmented or computationally demanding analyses, limiting their practical application for large-scale environmental monitoring. The significance of our work lies in developing MetaRanker, which overcomes these barriers by delivering a fast, accurate, and integrated metric of resistome risk. By simultaneously accounting for the abundance, mobility potential, and pathogenicity linkage of resistance determinants, MetaRanker enables a more realistic threat assessment. This tool empowers researchers and public health officials to track resistance hotspots, evaluate the impact of human activities such as waste disposal, and monitor the effectiveness of mitigation strategies, ultimately supporting data-driven decisions to curb the environmental spread of resistance.

## INTRODUCTION

Antibiotic resistance poses a critical global public health threat. The accumulation and widespread dissemination of antibiotic resistance genes (ARGs) across diverse environments substantially endanger both human and environmental health (1–4). The scale of this threat is underscored by estimates linking over 4 million annual deaths to antibiotic-resistant infections, with approximately 20% directly attributable to them—a figure projected to reach 8 million by 2050 (5). These infections complicate treatments, prolong hospitalizations, increase mortality, and disrupt agriculture and food safety, culminating in profound economic losses (6).

The One Health framework conceptualizes antibiotic resistance not merely as a human health issue, but as a complex challenge arising from the interconnectedness of environmental, animal, and human domains (7–10). Within this framework, the horizontal transfer of ARGs from environmental microbes to human pathogens via mobile genetic elements (MGEs) is a key dissemination pathway, enabling outbreaks through food chains and water bodies (11). Consequently, monitoring environmental ARGs is fundamental to developing targeted strategies that interrupt these transmission routes.

The public health risk of ARGs materializes primarily upon their transfer into pathogenic hosts, a process driven by horizontal gene transfer (HGT) mediated by MGEs (12). Plasmids, integrons, transposons, and insertion sequences facilitate the intra- and inter-cellular mobility of ARGs, allowing their dissemination across microbial communities and ecosystems (13, 14). When ARGs are harbored by pathogens, they compromise antibiotic efficacy, leading to treatment failure, prolonged illness, and increased healthcare costs (15, 16).

The collective repertoire of ARGs and their associated genetic elements within a microbiome, termed the resistome, is central to understanding the development and propagation of antibiotic resistance. The resistome encompasses both intrinsic resistance genes, which have evolved through long-term natural selection, and acquired resistance genes, whose mobilization and dissemination are frequently driven by anthropogenic antibiotic pressure. Together, these form a dynamic genetic reservoir that facilitates the horizontal transfer and evolution of resistance traits (17). The composition and structure of the resistome reflect the community’s capacity to acquire new resistance mechanisms, directly influencing the overall threat level. Thus, accurately profiling the resistome is crucial for assessing risks and informing the development of novel control strategies (18, 19).

Metagenomic sequencing has emerged as the gold standard for resistome characterization, offering broad coverage, high throughput, and non-targeted sensitivity (20, 21). It enables comprehensive, quantitative profiling of resistance types and abundances. Furthermore, assembling sequencing reads into contigs reveals the genomic context of ARGs, including co-localization with MGEs and virulence factors (VFs) on the same genetic element—a key indicator of dissemination potential, particularly with long-read technologies (22). However, current computational tools for translating this data into a quantitative risk assessment face significant limitations in accuracy and efficiency.

Existing methods, such as MetaCompare (23), ARGem (24), and ARG ranker (25), illustrate the current landscape and its shortcomings. MetaCompare, a pioneering tool for environmental resistome risk assessment, calculates a risk score based on the co-occurrence of ARGs, MGEs, and VFs on contigs. Its recent update, MetaCompare 2.0, separately evaluates environmental and human health risks (26). While valuable, its contig-based gene quantification overlooks sequencing depth, potentially compromising accuracy. ARGem utilizes both reads and contigs, aligning reads to ARG databases for quantification and annotating contigs for co-occurrence analysis. This approach should offer more accurate quantification but lacks a unified framework for quantifying overall sample risk and omits VFs (24). ARG ranker employs a decision tree to classify the risk levels of individual ARGs based on their enrichment, association with MGEs, and presence in pathogens such as ESKAPE (25). While effective for gene-centric risk grading, it does not provide a holistic, quantitative assessment of the antibiotic resistance risk posed by a metagenomic sample as a whole.

The limitations of these tools are rooted in the fundamental choices for quantifying risk elements (REs) from metagenomic data. Generally, abundance can be derived via: (i) aligning annotated components from contigs with sequencing reads; (ii) reads-based binning methods; or (iii) direct alignment of reads to reference databases. Critically, reads-based quantification methods are known to be more accurate than direct counting from contigs (27). Simultaneously, contigs provide indispensable information by revealing the upstream and downstream genetic structures of these elements, which is crucial for co-occurrence analysis (28). Therefore, an optimal framework must integrate the quantitative accuracy of reads with the contextual intelligence of contigs.

To bridge this gap, we present MetaRanker, a computational workflow designed for the comprehensive risk assessment of the environmental resistome. MetaRanker integrates the abundance of ARGs, MGEs, and VFs, calculated directly from sequencing reads for accuracy, with their co-occurrence structures on contigs. By unifying these aspects into a single (RI) and optimizing the computational process, MetaRanker achieves a holistic, precise, and efficient evaluation of antibiotic resistance risk in metagenomic samples.

## MATERIALS AND METHODS

### Overview of MetaRanker

**Fig 1:**
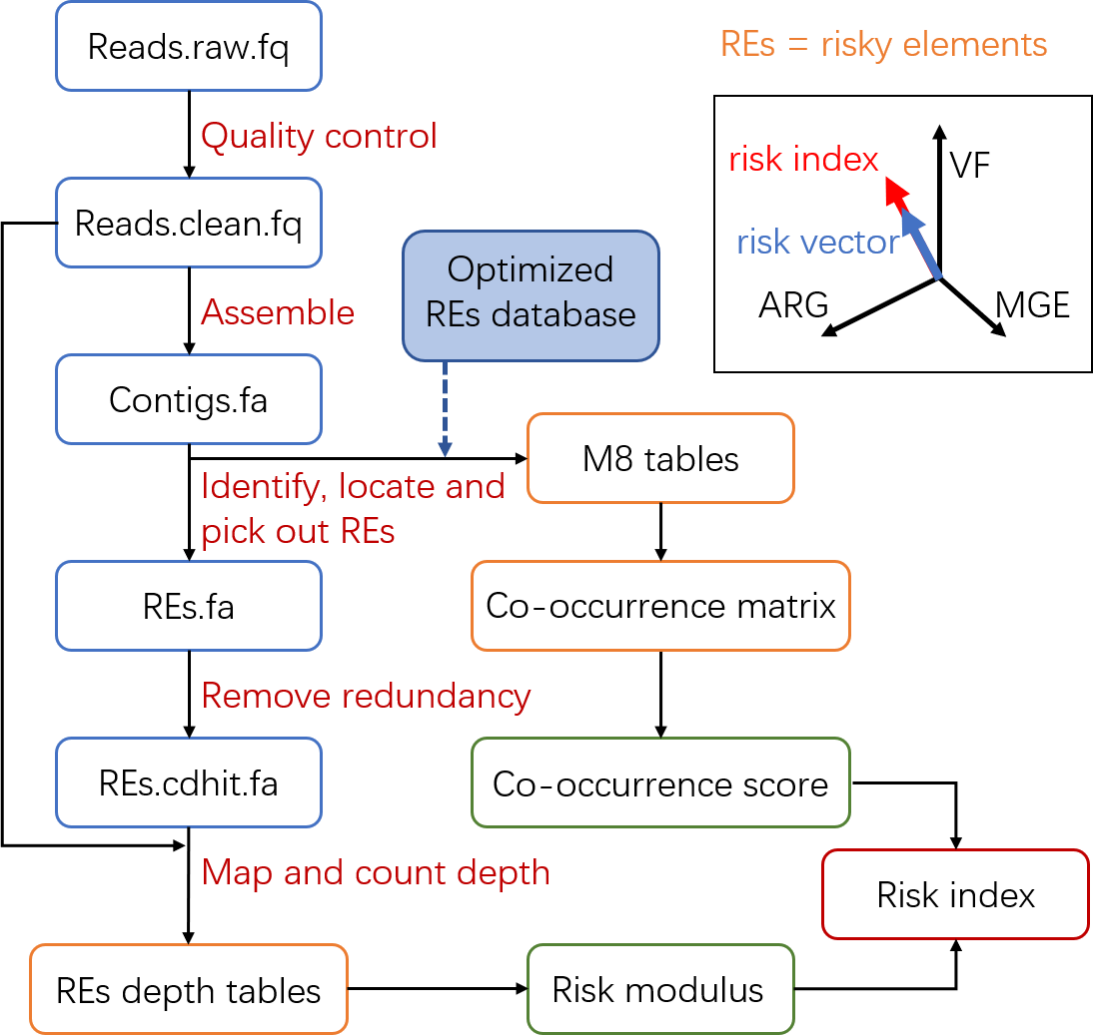
Schematic diagram of the MetaRanker computational workflow. Blue boxes denote sequence data, orange boxes represent tables or matrices, and green/red boxes indicate scalar values. The inset illustrates the sample risk vector within the 3D hazard space.

In MetaRanker, antibiotic resistance genes (ARGs), mobile genetic elements ( MGEs), and virulence factors (VFs) are collectively defined as risk elements (REs), which form the basis of the risk assessment. The computational workflow, illustrated in Fig. 1, proceeds as follows.

Quality-controlled metagenomic reads and assembled contigs serve as the input. Short reads (e.g., Illumina) are processed with Fastp (29) and assembled using Megahit (30) or other assembly tools, while long reads (e.g., Nanopore or PacBio) are quality-filtered with Chopper (31) and assembled with Flye (32). To ensure assembly quality, only samples yielding a minimum of 2,000 contigs, each longer than 500 bp, are retained for subsequent analysis.

Contigs are annotated against the RE databases using BLASTn (33). RE segments are extracted based on these annotations, and their physical co-localization on contigs is used to construct co-occurrence vectors and a sample-level co-occurrence matrix. Redundant RE segments are subsequently clustered at 85% identity using CD-HIT (34) to generate a non-redundant RE set. This set is aligned back to the quality-controlled reads using BWA (35) for short reads or Minimap2 (36) for long reads. The sequencing depth of each RE segment is calculated using SAMtools (37). The relative abundance of REs is then determined based on segment length and sequencing depth, forming a risk abundance matrix.

The quantitative model for antibiotic resistance risk assessment is defined as follows. For a contig C, its risk is represented by a vector:


(1) $$Q(C)=[q_{ARG}(C),q_{MGE}(C),q_{VF}(C)]$$

where $q_{ARG}\left(C\right),q_{MGE}\left(C\right),q_{VF}\left(C\right)$ denote the counts of ARG, MGE, and VF annotations on *C*, respectively.

For a given sample, the co-occurrence matrix R is constructed as an *m* × 3 matrix, where each row corresponds to a contig *C_i_* harboring at least one RE:


(2) $$R=\left[\begin{array}{c} q_{ARG}\left(C_{1}\right),q_{MGE}\left(C_{1}\right),q_{VF}\left(C_{1}\right),\;\\ q_{ARG}\left(C_{2}\right),q_{MGE}\left(C_{2}\right),q_{VF}\left(C_{2}\right),\\ \ldots \\ q_{ARG}\left(C_{m}\right),q_{MGE}\left(C_{m}\right),q_{VF}\left(C_{m}\right), \end{array}\right]$$

Here, *m* denotes the total number of RE-bearing contigs. This matrix captures the premise that the co-presence of ARGs, MGEs, and VFs in a sample signifies potential resistance risk, which is substantially heightened when these elements are physically colocalized on the same contig, facilitating coordinated transfer.

The co-occurrence score is defined as the total number of RE annotations divided by the number of RE-bearing contigs (*m*):


(3) $$cooccur\;score=\frac{\sum_{m}q_{ARG}\left(C_{i}\right)+\sum_{m}q_{MGE}\left(C_{i}\right)+\sum_{m}q_{VF}\left(C_{i}\right)}{m}$$

This score reflects the density of REs on risky contigs. A value closer to 1 indicates fewer co-occurrence events, while higher values suggest a greater probability of RE co-localization.

The aggregated base count (*b*_RE_) for each RE is calculated as the sum of the product of the length (*l*_RE_) and sequencing depth (*d*_RE_) of all its segments:


(4) $$b_{RE}=l_{RE}\times d_{RE}$$

The relative abundance (*d̄*_RE_) is then computed as the proportion of bases belonging to the RE in the total sequenced bases:


(5) $${\bar{d}}_{RE}=b_{RE}/\sum b_{reads}$$

where $\sum b_{reads}$ denotes the total number of bases in the quality-controlled reads.

The sample’s risk vector $\vec{r}$ is derived from the weighted relative abundances of the three REs:


(6) $$\vec{r}=\left[\sum {\bar{d}}_{ARG},\sum {\bar{d}}_{MGE},\sum {\bar{d}}_{VF}\right]\cdot \left[w_{ARG},w_{MGE},w_{VF}\right]$$

Fixed weighting factors ($w_{\text{ARG}}=5\times 10^{4}$, $w_{\text{MGE}}=1\times 10^{4}$, $w_{\text{VF}}=2\times 10^{4}$) were applied to the relative abundances of the three REs to standardize them to a comparable order of magnitude and to scale the majority of risk modulus values between 1 and 100. These weights were empirically determined based on the distribution of RE abundances in our in-house metagenomic data set (*n*= 103; Table S4). Specifically, we calculated the mean ($3.63\times 10^{-4}$, $2.50\times 10^{-3}$, $5.57\times 10^{-4}$) and median ($9.32\times 10^{-5}$, $7.80\times 10^{-4}$, $3.53\times 10^{-4}$) values for the aggregated relative abundances of ARGs, MGEs, and VFs, respectively. The final weighting factor for each RE was then set as the intermediate value between the reciprocals of its mean and median abundance.

The risk modulus is defined as the magnitude of the risk vector:


(7) $$risk\;modulus=\left|\vec{r}\right|=\sqrt{{\left(\sum {\bar{d}}_{ARG}\cdot w_{ARG}\right)}^{2}+{\left(\sum {\bar{d}}_{MGE}\cdot w_{MGE}\right)}^{2}+{\left(\sum {\bar{d}}_{VF}\cdot w_{VF}\right)}^{2}\#}$$

Finally, the comprehensive RI integrates both abundance and genetic context:


(8) RI=risk modulus×cooccur score

The RI thus provides a unified metric that reflects both the abundance of ARGs, MGEs, and VFs and their potential for co-transfer, offering a robust measure of the sample’s antibiotic resistance risk. The simultaneous detection of ARGs, MGEs, and VFs in an environmental sample signifies a potential risk for the emergence of drug-resistant infections, even in the absence of their physical co-localization on a shared replicon. Their simultaneous presence facilitates horizontal transfer and assembly in opportunistic pathogens.

### Database construction

The reference databases for RE annotation comprise the following: the Comprehensive Antibiotic Resistance Database (CARD) (38) for ARGs, the Virulence Factor Database (VFDB) (39) for VFs, and a consolidated MGE database incorporating ISfinder (40), TnCentral (41), INTEGRALL (42), and the PlasmidFinder database (43), thus covering insertion sequences, transposons, integrases, and plasmid replicons. To minimize redundancy, each database was first clustered using CD-HIT at 85% sequence identity and coverage. This threshold was empirically determined to balance annotation accuracy and computational efficiency (Table S7). Subsequently, BLASTn was employed to identify and remove sequences shared between different RE categories (e.g., VFs vs. ARGs, VFs vs. MGEs, ARGs vs. MGEs), thereby ensuring unambiguous classification and eliminating overlaps (Table S8), especially like multi-drug resistance plasmids in MGE database. The resulting integrated database is non-redundant both within and across RE categories, which enhances the accuracy and reliability of subsequent gene quantification and risk assessment. The final processed database has a total size of approximately 29.6 MB, reducing computational overhead while preserving annotation precision.

### Sequence searching and alignment strategies

The risk assessment workflow employs a multi-step alignment strategy using BLASTn, CD-HIT, and BWA/Minimap2. REs were first annotated directly on contigs using BLASTn (E-value < 10⁻⁴, identity ≥ 85%, coverage ≥ 75 bp), in which the gene prediction step was skipped, to improve the recall of non-coding sequences and simplify computation. Following annotation, RE sequences were extracted from the contigs. Redundant sequences were then clustered using CD-HIT (identity ≥ 85%, coverage ≥ 85%, precise mode) to generate a non-redundant RE set. Finally, this non-redundant set was aligned back to the quality-controlled sequencing reads using BWA (for short reads) or Minimap2 (for long reads) with default parameters to calculate coverage depth. This read-based alignment provides a precise measure of RE abundance for accurate quantitative risk assessment.

### Data sets, experiment, and statistical analysis

To comprehensively evaluate MetaRanker, we employed a combination of simulated and real metagenomic data sets. Our analysis included 20 *in silico* mock samples for benchmarking (Table S8). Furthermore, we integrated 103 metagenomic samples from our previous studies (Table S4) with 250 publicly available samples, comprising 240 short-read and 10 long-read data sets (Tables S5 and S6, respectively). All samples were categorized based on their environmental origin.

Subsets of these data were used for specific validation purposes. From the 240 public short-read samples, nine representative samples (Table S2) were selected to investigate the influence of sequencing depth on risk assessment. Additionally, a distinct set of 48 samples, spanning a spectrum of risk levels (Table S3), was used for a comparative analysis against MetaCompare 2.0 and to validate MetaRanker’s abundance calculation and contig assembly approaches. All data processing, statistical analyses, and visualizations were performed using Python.

### Benchmarking with *in silico* samples

To validate the quantitative performance of MetaRanker in resistome risk assessment, we constructed four distinct sets of *in silico* samples, each comprising five samples simulating a gradient of environmental contamination. This gradient was created by mixing sequencing reads from high-risk and low-risk sources at defined ratios of 0:100, 25:75, 50:50, 75:25, and 100:00 (high-risk: low-risk). This design was implemented across the four distinct sample sets to validate MetaRanker under different scenarios. Set 1 comprised mixtures of clean reads from a high-risk clinical sample (SRR22519794, feces from an antibiotic-treated patient) and a low-risk environmental sample (QLFW, freshwater from Qinghai Lake). For Set 2, we generated reads *in silico*: the high-risk component (HRG, high-risk genome) was derived from genomes of ESKAPE pathogens and multidrug-resistant plasmids (Table S1), while the low-risk component (LRG, low-risk genome) originated from common soil microorganisms (Table S1). Set 3 was designed to hybridize real clinical and simulated environmental data by mixing clean reads from the high-risk sample SRR22519794 with the *in silico* generated LRG reads. Conversely, Set 4 combined the *in silico* generated HRG reads with clean reads from the low-risk environmental sample QLFW, creating a hybrid of simulated clinical and real environmental data.

The HRG and LRG reads were generated using a standardized *in silico* simulation pipeline. First, the reference genomes were fragmented into contigs with a target length of 5,000 bp (following a normal distribution N [5,000, 1,000], minimum length 1,000 bp), introducing 50 bp gaps between adjacent contigs to prevent mis-assembly into longer sequences. These contigs were then used as templates to generate 150 bp reads (N [150, 3], length range 144–156 bp). Reads were uniformly sampled from all possible positions across all contigs, producing approximately 60 million reads (~9 Gb of data) for each HRG or LRG sample. The simulated reads from all sample sets were assembled into contigs using MegaHit, and the resulting assemblies were assessed by both MetaRanker and MetaCompare 2.0.

**Fig 2:**
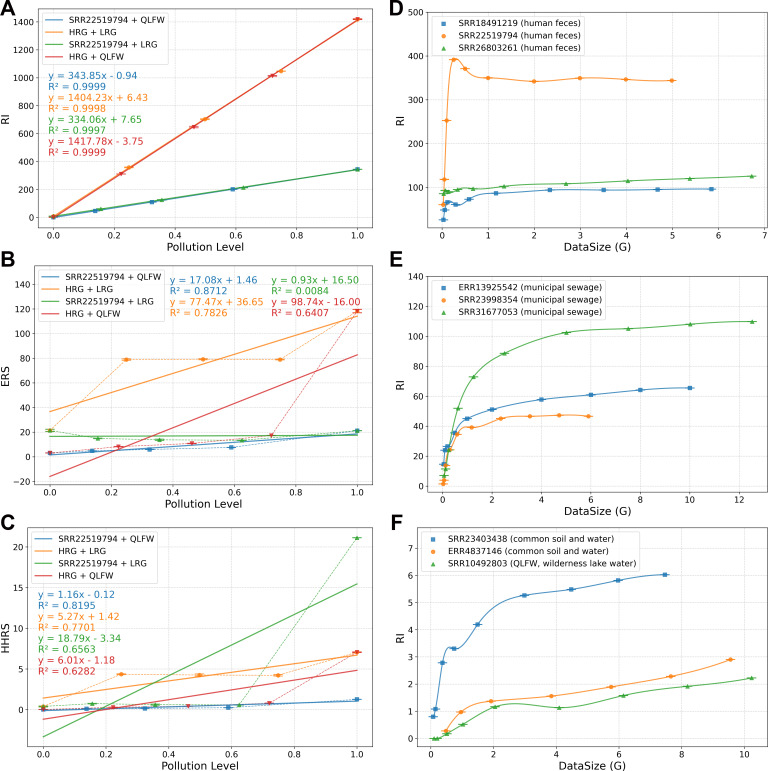
Benchmarking analysis using *in silico* samples and the impact of sequencing depth. (**A–C**) Risk assessment results of four sets of simulated samples with varying contamination levels. (**A**) Risk index (RI) from MetaRanker. (**B**) Ecological risk score (ERS) from MetaCompare 2.0. (**C**) Human health risk score (HHRS) from MetaCompare 2.0. (**D–F**) The influence of sequencing data volume on the RI assessed by MetaRanker across different sample types. (**D**) Human feces samples. (**E**) Municipal sewage samples. (**F**) Common soil and water samples. The risk index (RI) is calculated as the product of the risk modulus (weighted sum of ARG, MGE, and VF abundances) and the co-occurrence score. Weighting factors are applied to balance the contributions of each RE type.

In parallel, to evaluate the impact of sequencing depth on MetaRanker’s risk assessment, we generated subsamples from nine metagenomic samples (Table S2) by randomly selecting 1%, 2%, 5%, 10%, 20%, 40%, 60%, and 80% of the reads, yielding a series of data sets (Fig. 2). These subsamples were assembled using MegaHit and subsequently analyzed by MetaRanker. To ensure the reproducibility and robustness of all random sampling and sequence generation steps, we performed three technical replicates for each, using fixed random seeds of 1, 2, and 3.

### Comparison to MetaCompare 2.0 on real samples

To evaluate MetaRanker’s performance on real metagenomic data, we compared its risk assessments against those generated by MetaCompare 2.0 using 48 publicly available samples representing diverse environments (Table S3). We assessed the agreement between the tools through linear regression and intraclass correlation coefficient (ICC) analysis, while also evaluating discriminatory power via analysis of variance. To enable direct comparison, the ecological and human health risk scores from MetaCompare 2.0 were proportionally scaled so that their overall average matched that of MetaRanker’s RI. Furthermore, we compared computational efficiency by measuring runtime under identical hardware configurations (32 CPU cores, 128 GB RAM), with both tools executing in parallel using all available threads.

### Validation of quantification method

To evaluate the quantification accuracy of MetaRanker’s read-based approach, which calculates the proportion of bases in clean reads belonging to REs to control for biases from variable read lengths, we performed comparative analyses against two conventional abundance estimation methods.

First, we compared MetaRanker’s risk vector values against gene-centric abundances derived from open reading frame (ORF) prediction. For a representative subset of samples across environmental categories, contigs were uniformly assembled and processed with Prodigal (44) to predict genes, which were subsequently dereplicated. The resulting non-redundant gene sets were annotated against the same RE databases used in MetaRanker via BLASTn. Gene depths were then obtained by aligning these gene sets back to the original sequencing reads, and abundances were calculated as reads per million (RPM) and reads per kilobase per million (RPKM), defined as:


(9) $$RPM=d_{gene}/\left(\sum d_{total\;reads}\times 10^{-6}\right)$$

(10) $$RPKM=\frac{d_{gene}}{l_{referenced\;gene}\times 10^{-3}}/\left(\sum d_{total\;reads}\times 10^{-6}\right)$$

Second, we benchmarked MetaRanker against the read alignment-based strategy employed by ARGem. Here, Bowtie2 (45) was used to directly align quality-controlled reads to the indexed MetaRanker RE databases. The depth of each RE was extracted using SAMtools and normalized as bases per million total bases (BPM), a length-aware abundance metric calculated as:


(11) $$BPM=d_{target}\times l_{target}/\sum \left(d_{reads\times }\times l_{reads}\right)\times 10^{6}$$

The three components of MetaRanker’s risk vector were then separately compared to the corresponding RE abundances derived from the RPM, RPKM, and BPM methods. Consistency was assessed using linear regression and the ICC. Prior to ICC calculation, abundance values from all alternative methods were proportionally scaled so that the global mean abundance of each RE type matched that of the corresponding MetaRanker risk vector component.

### Impact of assembly tools on risk assessment

To evaluate the robustness of MetaRanker to variations in metagenome assembly, we generated contigs from the same set of sequencing reads using two distinct assemblers: the resource-efficient Megahit and the more computationally intensive MetaSpades (46). The resulting contig sets were then processed independently through the MetaRanker pipeline. We assessed the consistency of the computed risk indices (RIs) between the two assembly methods using linear regression and ICC analysis.

### MetaRanker validation on diverse environmental data sets

To comprehensively evaluate MetaRanker’s ability to discriminate antibiotic resistance risks across environments, we applied it to an integrated data set of 103 in-house metagenomic samples (Table S4) and 240 publicly available short-read samples (Table S5). We assessed the significance of risk differences between pre-defined sample groups by first testing the distribution properties of the RI values using the Shapiro-Wilk test (normality) and Levene’s test (homogeneity of variance). As the data violated parametric assumptions, inter-group differences were subsequently analyzed using the non-parametric Mann-Whitney *U* test, with Bonferroni correction applied to account for multiple comparisons.

For intuitive visualization of risk profiles, we projected the samples into a three-dimensional hazard space defined by the three components of the risk vector (ARG, MGE, and VF abundance). In this space, the position of a sample reflects its risk composition, while the point size is proportional to its co-occurrence score, thereby highlighting samples with frequent genetic co-localization of REs. Furthermore, to facilitate the biological interpretation of high-risk samples, MetaRanker can extract and output contig sequences exhibiting co-linearity of multiple REs, which can be visualized using the companion tools provided in the code repository.

### Identification of high-risk REs

To identify REs posing the greatest potential threat to human health, we conducted a comprehensive analysis of their abundance, co-occurrence frequency, and prevalence across all 343 short-read metagenomic samples. Co-occurrence frequency was calculated from the co-occurrence matrix as the number of instances in which a specific RE was found on the same contig as any other RE, while prevalence was defined as the frequency of its detection across samples. REs exhibiting concurrently high values across all three metrics were classified as high risk. These high-risk elements were subsequently analyzed and categorized by their functional type (ARGs, MGEs) to elucidate predominant risk patterns.

## RESULTS

### MetaRanker accurately quantifies resistome risk in simulated samples

The RI computed by MetaRanker integrates both the abundance and co-occurrence of REs. The relative abundances of ARGs, MGEs, and VFs are weighted to standardize their contributions to the risk modulus, ensuring that each RE type is balanced in scale and biological relevance. The weighting factors ($w_{\text{ARG}}=5\times 10^{4}$, $w_{\text{MGE}}=1\times 10^{4}$, $w_{\text{VF}}=2\times 10^{4}$) were empirically determined based on the distribution of RE abundances in an in-house metagenomic data set (*n* = 103; see Materials and Methods for details). This weighting scheme scales the majority of risk modulus values between 1 and 100, facilitating intuitive interpretation and comparison across samples.

MetaRanker demonstrated precise quantification of contamination levels and resistome risk in benchmarking experiments. As shown in Fig. 2A through C, across four distinct types of simulated samples contaminated with antibiotic-resistant bacteria, the RI generated by MetaRanker showed a strong positive correlation with the degree of contamination (Fig. 2A, *R*² ≈ 1). In contrast, risk scores produced by MetaCompare 2.0 displayed limited correlation with contamination levels in both its ecological risk (Fig. 2B, *R*² ≤ 0.87; range: 0.01–0.87) and human health risk (Fig. 2C, *R*² ≤ 0.82; range: 0.63–0.82) scores. Analysis of sequencing data characteristics (Table S9) revealed that MetaCompare 2.0’s reliance on contig-based quantification and normalization by contig count substantially influenced its risk scores, consequently limiting its ability to differentiate between high- and low-risk mixed samples. Unlike this approach, MetaRanker employs read-based quantification normalized by sequencing depth, which is essential for accurate gene abundance estimation in high-throughput sequencing data, thereby producing risk assessments that better reflect actual sample conditions.

Evaluation of subsampled data sets (Fig. 2D through F) revealed that RI values stabilized with increasing sequencing depth, with saturation points varying across environmental types due to differences in microbial diversity. Based on these trends, we estimate the minimum sequencing requirements for MetaRanker as follows: approximately 0.5 Gb for human gut/fecal samples, 3–5 Gb for municipal wastewater, and 5–10 Gb or more for natural environmental samples. These thresholds represent the sequencing depths at which genome coverage becomes sufficient for comprehensive RE detection and stable risk assessment.

### MetaRanker demonstrates superior discriminatory power and computational efficiency on real samples

**Fig 3:**
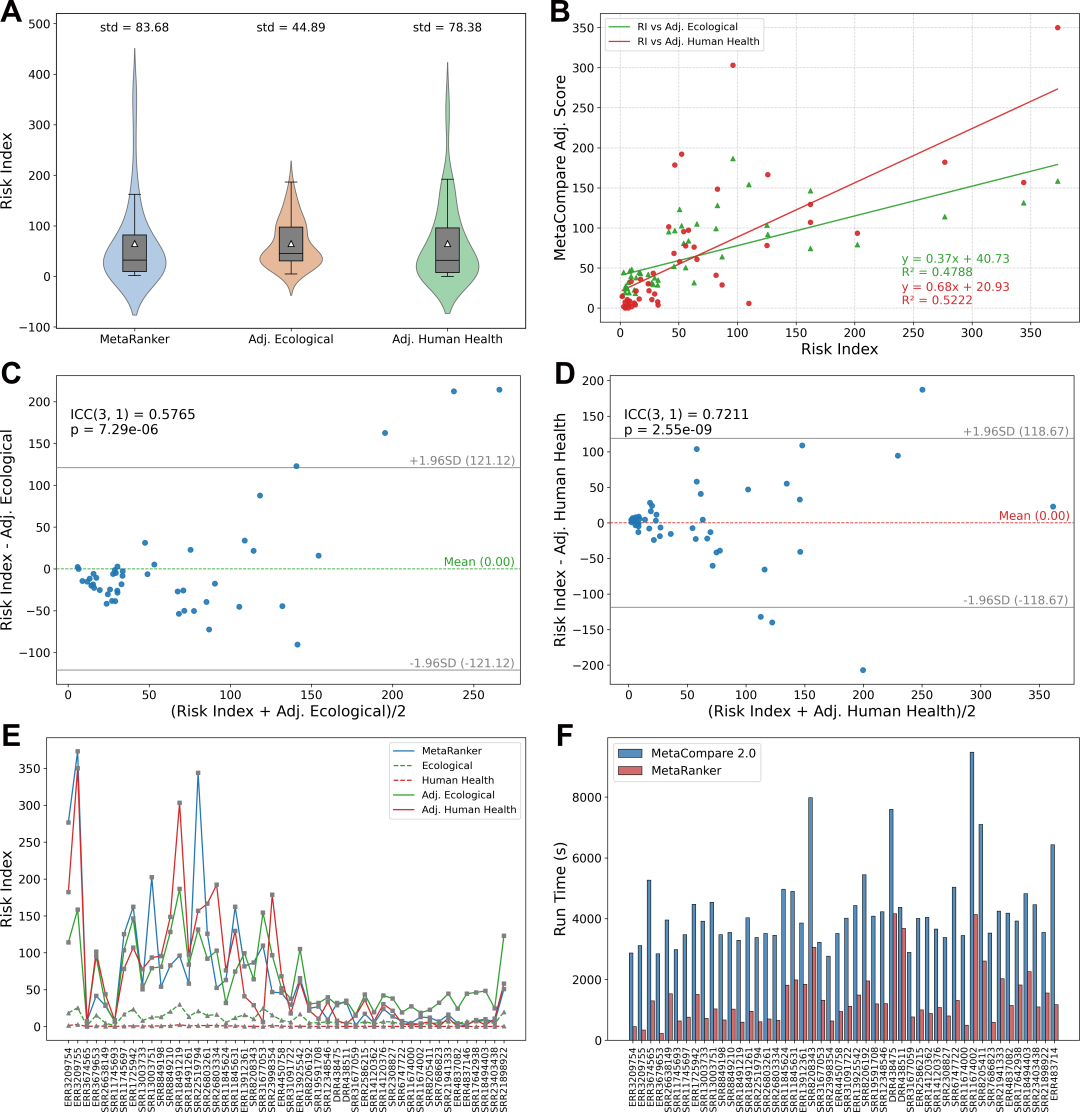
Comparison of resistance risk results between MetaRanker and MetaCompare 2.0. (**A**) Violin plots and standard deviation of RI, ecological risk score, and human health risk score. The risk scores from MetaCompare 2.0 were adjusted to match the same level as MetaRanker’s RI (with equal average values). (**B**) Linear regression analysis of RI and the scaled ecological risk score and human health risk score. (**C, D**) Consistency analysis of RI and the scaled ecological risk score and human health risk score, represented by the Bland-Altman plot. (**E**) Line chart of RI, ecological risk score, and human health risk score. (**F**) Bar chart of runtime for MetaRanker and MetaCompare 2.0. This analysis used 48 representative samples (see Table S3).

MetaRanker’s risk assessments show measurable differences from those of MetaCompare 2.0 and exhibit enhanced ability to discriminate between samples. As shown in Fig. 3B through E, MetaRanker’s RI displays moderate correlation with the adjusted environmental risk score from MetaCompare 2.0 (*R*² = 0.4788, ICC(3,1) = 0.5765), while showing stronger agreement with the human health risk score (*R*² = 0.5222, ICC(3,1) = 0.7211). This likely reflects the closer biological relevance of the VFDB to human health outcomes. The VFDB was originally constructed based on 16 major human bacterial pathogens, establishing an intrinsic focus on factors contributing to human infection and disease (47).

Although the overall distributions of the three risk metrics are similar after mean scaling (Fig. 3A), MetaRanker’s RI shows a wider dispersion, as indicated by its larger standard deviation. A paired F-test confirmed significantly greater variance in RI compared to the environmental risk score (*P* = 1.88 × 10⁻⁵), but not against the human health risk score (*P* = 0.3277). Furthermore, a regression slope below 1 and Bland–Altman analysis, revealing increasing deviation with higher risk values, collectively suggest that MetaRanker provides greater dynamic range in risk quantification. These patterns support its improved ability to distinguish samples with varying risk levels, attributable to its more accurate read-based quantification and its integrative risk assessment framework (see Discussion).

In cases where MetaRanker and MetaCompare 2.0 produced divergent risk estimates, we attribute the discrepancy primarily to the more precise gene quantification achieved by MetaRanker, which better approximates true biological risk. In addition to accuracy gains, MetaRanker offers substantial computational advantage, requiring only 20–50% of the runtime of MetaCompare 2.0 (Fig. 3F). This efficiency is achieved despite processing more nucleotide sequences, resulting from database optimization and strategically streamlined alignment procedures.

### MetaRanker’s accurate quantification of REs

To evaluate the accuracy of MetaRanker’s read-based quantification of risk elements (REs), we compared its risk vector values against abundances derived from two conventional approaches: (i) gene prediction from contigs followed by read mapping (yielding RPM and RPKM values) and (ii) direct read alignment against reference databases (yielding BPM values). As shown in Fig. S1A through C, the abundance estimates for ARGs, MGEs, and VFs from MetaRanker showed strong concordance with those from the alternative methods. Specifically, the coefficients of determination (*R*²) were predominantly greater than 0.8306, and the intraclass correlation coefficients (ICC(3,1)) largely exceeded 0.95 (*P* < 10⁻⁴). The only exceptions were observed for VF abundances based on BPM, which exhibited lower agreement and a limited number of samples analyzed using RPKM.

The high degree of correlation and consistency confirms that MetaRanker’s quantification strategy performs on par with well-established gene- and read-based methods. The lower agreement for VFs with BPM may stem from the presence of long sequences in VFDB, which can be annotated differently by BLASTn on contigs versus direct read alignment. Similarly, the occasional discrepancy with RPKM may arise because RPKM uses the full reference gene length in its denominator, including regions not covered by alignments, thereby introducing normalization artifacts not present in MetaRanker’s segment-based depth calculation.

### MetaRanker’s risk assessment is robust to assembly tool selection

MetaRanker demonstrated consistent performance regardless of the metagenome assembler used, showing strong agreement between contigs generated by Megahit and the more computationally intensive MetaSPAdes, for which 29 of 48 samples were successfully assembled. As shown in Fig. S1D and E, the RI values derived from Megahit and MetaSPAdes contigs were highly concordant, with a linear regression slope near y = x and a coefficient of determination (*R*²) approaching 1. Intraclass correlation analysis further confirmed nearly perfect agreement (ICC(3,1) ≈ 1, *P* ≈ 0). Bland–Altman analysis indicated a slight systematic elevation in RI from Megahit relative to MetaSPAdes, though with minimal random error. We hypothesize that this minor deviation may stem from the generally longer contigs produced by Megahit (Fig. S1F and G), which could facilitate the annotation of a greater number of REs by BLASTn (Fig. S1H). Nonetheless, these results affirm that MetaRanker’s risk assessment remains highly consistent across different assembly tools.

### MetaRanker effectively discriminates resistome risks across diverse environmental samples

**Fig 4:**
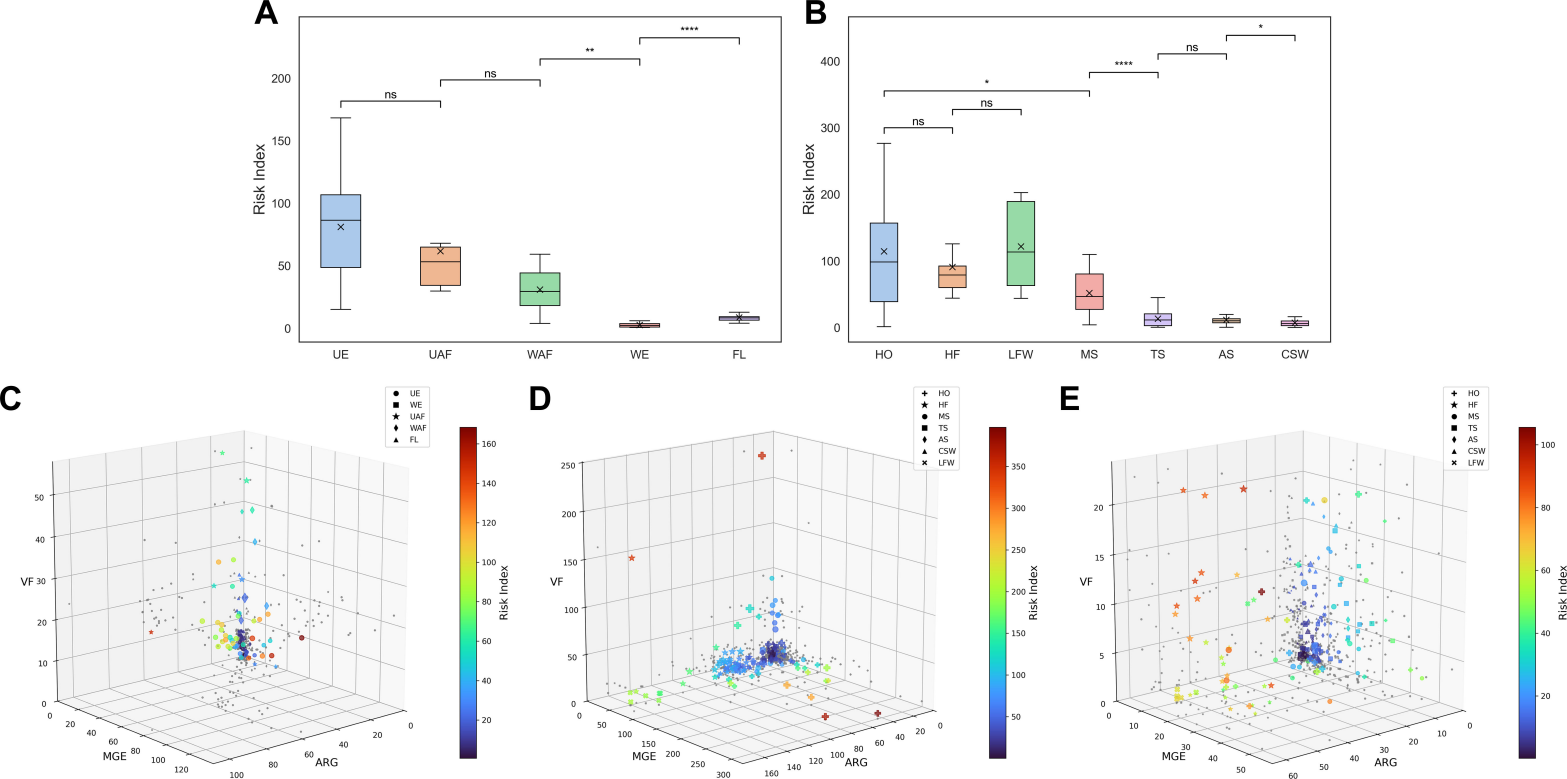
MetaRanker effectively discriminates antibiotic resistome risks across environmental samples. (**A**) Distribution of risk index (RI) for 103 in-house metagenomic samples. UE: urban environment (*n* = 39); WE: wilderness environment (*n* = 8); UAF: urban animal feces (*n* = 7); WAF: wild animal feces (*n* = 8); FL: farmland (*n* = 41). (**B**) RI distribution for 240 public short-read metagenomic samples. HO: hospital (*n* = 46); HF: human feces (*n* = 39); MS: municipal sewage (*n* = 35); TS: treated sewage (*n* = 35); AS: activated sludge (*n* = 29); CSW: common soil and water (*n* = 41); LFW: livestock feces and wastewater (*n* = 12); bird feces (*n* = 3, omitted from visualization). Inter-group significance was assessed using the Mann-Whitney *U* test with Bonferroni correction (ns, not significant; *, *P* < 0.05; **, *P* < 0.01; ***, *P* < 0.001; ****, *P* < 0.0001). (**C–E**) Three-dimensional hazard space visualization. Sample position is determined by the risk vector (ARG, MGE, and VF abundance), distance from the origin corresponds to the risk modulus, point size reflects the co-occurrence score, and color represents the RI. (**C**) 103 in-house samples. (**D**) 240 public samples. (**E**) A subset of 183 lower-risk public samples.

MetaRanker successfully distinguished resistome risks across a range of environmental settings. As illustrated in Fig. 4A and B, urban samples (UE) exhibited a markedly higher mean risk index (RI = 81.01) compared to wilderness samples (WE, RI = 2.60; adj. *P* < 10^−4^), reflecting greater antibiotic resistance transmission potential in urban environments, likely due to higher population density, frequent antibiotic use, and wastewater discharge. Hospital samples (HO) showed the highest mean RI (114.51), significantly exceeding that of municipal sewage (MS, RI = 51.82; adj. *P* = 0.0025), underscoring their role as key reservoirs and hotspots for resistance gene dissemination. Wastewater treatment effectively reduced resistome risk, with RI declining from 51.82 (influent, MS) to 13.23 (effluent, TS; adj. *P* < 10⁻⁴). However, residual risk in treated effluent remained substantially elevated over natural background levels (e.g., WE: 2.60), highlighting the continued environmental release of resistance determinants and the need for advanced treatment and monitoring.

No significant difference was observed between human feces (HF) and hospital samples (adj. *P* = 1.0), suggesting a shared resistome pool between clinical and community settings. Livestock feces and wastewater (LFW) showed a trend toward higher RI than human feces (adj. *P* = 1.0), indicating a non-negligible contribution of animal agriculture to environmental resistance. Mitigation measures such as improved antibiotic stewardship and alternative disease management could help reduce this load. Activated sludge (AS) exhibited RI levels comparable to treated sewage (adj. *P* = 1.0) but significantly higher than common soil and water samples (CSW; adj. *P* = 0.0283), supporting careful consideration of its disposal or agricultural use.

Visualization of sample risks in the 3D hazard space (Fig. 4C through E) reinforced these trends. Samples with higher RE abundance were positioned farther from the origin, reflecting greater risk moduli, while larger point sizes—indicating higher RE co-occurrence frequency—highlighted samples with increased potential for HGT. Together, RE abundance and co-occurrence patterns provide complementary insights into resistome risk: abundance reflects the load of resistance and virulence determinants, whereas co-occurrence signals their mobilization potential and ecological persistence.

### MetaRanker supports risk assessment of third-generation sequencing data

MetaRanker maintains full compatibility with long-read sequencing technologies, accurately quantifying risk elements (REs) and calculating risk modulus from third-generation sequencing data (Table S6). The longer contigs typical of assemblies derived from such data facilitate the detection of a greater number of co-localized REs, resulting in systematically higher co-occurrence scores and, consequently, elevated risk indices (RIs) for these samples. Importantly, the computed RIs align with the expected risk profiles based on sample origin and environmental context, confirming the biological relevance of MetaRanker’s assessments across sequencing platforms.

### Identification of high-risk risk elements

**Fig 5:**
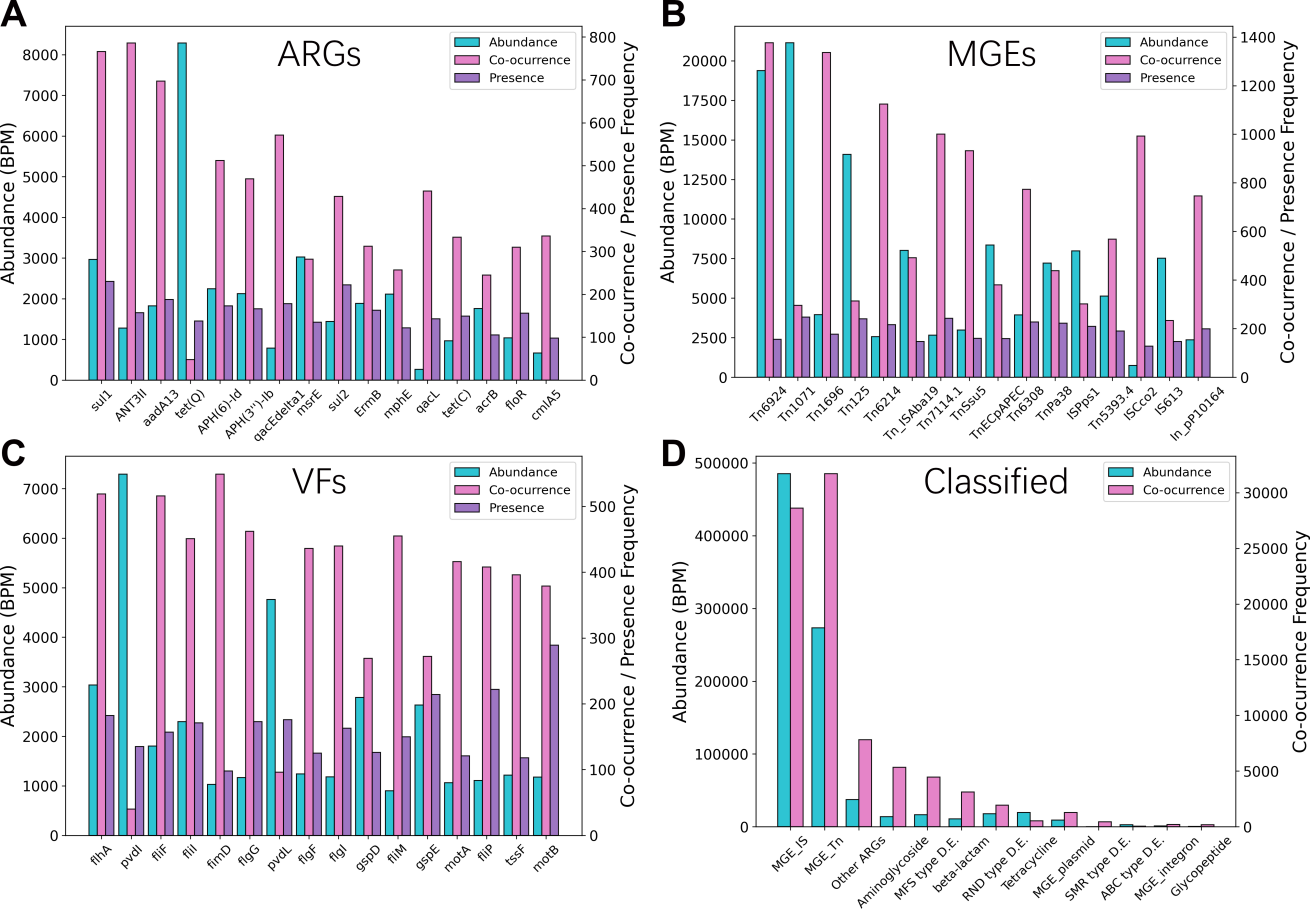
Statistics of high-risk REs. (**A–C**) Top 16 ARGs, MGEs, and VFs with high abundance, co-occurrence frequency, and presence frequency in 343 samples. (**D**) Classification statistics of ARGs and MGEs abundance and co-occurrence frequency.

Analysis of the abundance, co-occurrence frequency, and prevalence of REs across 343 short-read samples revealed distinct risk patterns (Fig. 5). Aminoglycoside, sulfonamide, and tetracycline resistance genes demonstrated notably high abundance and co-occurrence frequency, being detected in over 40% of samples (Fig. 5A). This pattern likely reflects the extensive clinical and agricultural use of these broad-spectrum antibiotics, which has driven the selection and enrichment of corresponding ARGs, often linked with enhanced mobility and multi-resistance phenotypes.

Among mobile genetic elements, transposons and insertion sequences predominated (Fig. 5B), exhibiting higher overall abundance than ARGs and virulence factors. Their prevalence underscores their role as key facilitators of resistance and virulence gene transfer. Even when not directly carrying ARGs or VFs, the high abundance of these MGEs establishes a permissive genetic background that potentially accelerates the dissemination of resistance determinants.

Virulence factors were predominantly associated with the genus *Pseudomonas* (Fig. 5C), an aerobic and metabolically versatile opportunistic pathogen commonly found in diverse environments, including wastewater, the primary source of most samples analyzed. A substantial proportion of these virulence genes encoded carrier proteins and flagellar components, with additional high-risk VFs comprising secretion system and cell wall-anchored proteins from other human pathogens such as *Shigella*, *Clostridium*, and *Acinetobacter*. This pattern aligns with the human-health-centric composition of the VFDB and underscores the clinical relevance of the detected high-risk VFs.

The characteristically higher abundance and co-occurrence frequency of insertion sequences and transposons relative to ARGs informed the weighting scheme in MetaRanker, justifying the relatively lower weight assigned to MGEs in the risk index calculation.

## DISCUSSION

### Advantages and significance

Environmental antibiotic resistance represents a critical global health challenge, driving increased research focus on resistome monitoring. The expanding use of metagenomic sequencing has led to rapidly growing data sets, creating a pressing need for standardized tools to evaluate and compare resistome risks across samples (48). However, processing such voluminous and complex data in an efficient, automated, and integrated manner remains a substantial challenge. To address this, we developed MetaRanker—a user-friendly, locally deployable computational pipeline for environmental resistome risk assessment. MetaRanker introduces a novel integrated framework that combines the abundance of risk elements (REs) with their co-occurrence structures into a unified RI. It supports both short- and long-read sequencing data and achieves high computational efficiency, overcoming key limitations of existing tools such as MetaCompare and ARGem.

MetaRanker demonstrated superior discriminatory power, as evidenced by the wider dispersion of its risk index (RI) values compared to those from MetaCompare 2.0 (Fig. 3A and B). While this primarily stems from its more accurate, read-based quantification capturing a broader dynamic range of risk, two key methodological features of our framework further contribute to this enhanced differentiation. First, the application of fixed, empirically determined weights to the abundances of ARGs, MGEs, and VFs ensures balanced contributions but can also amplify differences between samples with distinct risk element compositions. Second, the integrative calculation of RI—multiplying the weighted abundance modulus by the co-occurrence score—means that samples with similar total abundance but differing genetic linkage patterns receive distinct scores. In contrast, tools relying on contig-count normalization or separate risk scores may compress this variability. Thus, the observed dispersion is not merely a statistical artifact but a reflection of MetaRanker’s more sensitive and biologically integrative risk assessment model.

MetaRanker enables accurate, sensitive, and rapid discrimination of resistome risks across samples and includes built-in visualization capabilities. For instance, it identifies and visualizes contigs carrying multiple co-localized REs (see Fig. S2 and Supplementary Information), allowing researchers to investigate potential horizontal gene transfer events and co-pathogenicity associations.

The pipeline employs a length-aware, read-based quantification approach. While this improves accuracy, it also means that host depletion may relatively increase RE abundance and elevate RI values. Thus, to ensure cross-study comparability, consistent pre-processing and quality control procedures must be applied to all samples.

Notably, unlike ARG ranker (which categorizes risk levels) or MetaCompare (which focuses on risk-contigs), MetaRanker incorporates all contigs containing MGEs or VFs, even in the absence of ARGs, into its risk model. This design acknowledges that MGEs and VFs contribute to resistance risk even when not physically linked to ARGs, providing a more holistic risk profile that captures both direct genetic linkages and potential resistance-enhancing contexts.

### Applications in environmental and clinical monitoring

MetaRanker’s RI offers a practical indicator for environmental surveillance and evidence-based policy-making. Our analyses indicate elevated risks in hospitals, human feces, and urban sewage, underscoring the need for treatment before environmental discharge. MetaRanker can serve as a monitoring tool for evaluating the efficacy of waste treatment processes and for tracking resistance trends in diverse ecosystems, including rivers (49), soils (50), and marine environments (51), enabling identification of resistance hotspots and informing targeted management strategies.

In wastewater treatment plants, routine application of MetaRanker can help optimize operational parameters, enhance ARG removal efficiency, and mitigate the environmental impact of treated effluent (52–54). In clinical settings, profiling patient gut or fecal samples with MetaRanker could aid in diagnosing resistant infections, guiding personalized therapy, and reducing intra-hospital transmission of resistant strains (55–57).

### Validation and robustness

MetaRanker has been validated extensively on 353 real-world environmental samples spanning hospitals, feces, sewage, river water, and agricultural soils. It produces consistent results under fixed parameter settings and shows strong agreement with standard gene quantification methods (e.g., RPM, RPKM, BPM). Its outputs also align with experimental findings from our prior studies on wild bird feces (58–60).

The tool is fully compatible with third-generation sequencing data. Its read-based quantification minimizes platform-specific bias, though longer reads naturally yield higher co-occurrence scores due to improved contiguity. Despite this, RI values from long-read data remain biologically consistent with sample origins. However, due to systematic differences in co-occurrence scoring and library preparation, we do not recommend comparing samples derived from different sequencing technologies.

Furthermore, MetaRanker’s four-step quantification workflow—annotation, selection, redundancy removal, and alignment—has been verified as robust. Its results are also stable across common assembly tools (e.g., Megahit, MetaSPAdes). Nevertheless, we advise using consistent sequencing and pre-processing methods within a given comparative study to avoid technical confounders.

### Implications for antibiotic stewardship

Analysis of high-risk REs across samples revealed that aminoglycoside, sulfonamide, and tetracycline resistance genes are among the most abundant and most frequently co-occurring ARGs—a pattern consistent with the widespread clinical and agricultural use of these drug classes (61). This correlation underscores how human antibiotic practices shape the environmental resistome. To curb the proliferation of these ARGs, improved antibiotic stewardship and waste management are urgently needed, along with continued monitoring of these high-priority resistance determinants in environmental settings.

### Limitations and future work

While MetaRanker provides an efficient solution for assessing drug-resistant infection risk in environmental metagenomes, several design choices entail certain limitations. The tool prioritizes accurate categorization of risk elements (REs) over precise functional annotation of individual genes. To optimize computational efficiency, we applied aggressive sequence clustering (CD-HIT, 85% identity) and removed overlapping entries across the ARG, MGE, and VF databases. These steps reduce sequence diversity and may obscure the detection of closely related variants or elements with multifunctional roles (e.g., a gene that could be annotated as both an ARG and a VF). However, this does not compromise risk assessment accuracy, as the read mapping threshold aligns with the clustering criteria. Moreover, the primary goal of MetaRanker is to provide a robust and comparable risk index, not to deliver exhaustive gene-level annotation. Therefore, these trade-offs are acceptable for the intended purpose. Nevertheless, researchers requiring granular functional annotation of REs should consider alternative tools.

The weighting of REs in the risk modulus represents another consideration. Initial batch-specific normalization limited cross-study comparability. We thus established fixed weights ($w_{\text{ARG}}=5\times 10^{4}$, $w_{\text{MGE}}=1\times 10^{4}$, $w_{\text{VF}}=2\times 10^{4}$) based on the abundance distribution of 103 in-house samples, which were subsequently validated on an independent public data set (*n* = 240). The distribution of weighted RE abundance (${\bar{d}}_{RE}\times w_{RE}$, RE = ARGs, MGEs, or VFs) shows no significant difference between these two datasets (independent samples *t*-test, p_ARG_ = 0.0688, adj. p_MGE_ = 0.6264, p_VF_ = 0.7098); meanwhile, the majority of risk modulus values was scaled between 1 and 100 as expected (Fig. S3). These empirical weights reflect the relative health threats posed by the three RE categories and can be adjusted by users. Future versions could benefit from machine learning-driven weight optimization on expanded data sets.

Unlike MetaCompare 2.0 (26), MetaRanker does not distinguish between ecological and human health risks. Although implementing such a distinction—e.g., by partitioning VFDB by host type—is technically feasible, it would reduce database comprehensiveness and computational efficiency. More importantly, such a separation contradicts the One Health perspective, which recognizes the interconnectedness of human, animal, and ecosystem health (11, 62). Furthermore, accurate taxonomic binning in metagenomes—particularly for complex environments like soil—remains challenging (63), and species-based risk classification would substantially increase sequencing requirements (1.5–5,000 Gb/sample) compared to MetaRanker’s current needs (0.5–10 Gb/sample). Thus, we deliberately retained an integrated risk assessment approach to maintain practicality for long-term environmental monitoring.

Several promising directions exist for future development:

- Refined RE weighting: Incorporating biological context—such as distinguishing conjugative vs. non-conjugative plasmids, or chromosomal vs. extrachromosomal ARGs—could improve risk interpretation. RE-specific weights could be assigned based on co-occurrence patterns (Fig. 5) or adopting ARG risk grading schemes (25).
- Host microbiome context: Integrating microbial host risk, such as the abundance of ESKAPE pathogens (64) or species strongly associated with ARG carriage (65), could enhance risk prediction, though this would require expanded database infrastructure.
- Pathogenicity database enhancement: Augmenting VFDB with ARG-associated sequences and ESKAPE-related virulence markers could improve pathogenicity risk profiling.
- Metagenome-assembled genomes (MAGs): Utilizing MAGs instead of contigs for co-occurrence analysis would provide more complete genetic context, though it would require adjustments to co-occurrence score calculations. We plan to continue validating and expanding MetaRanker’s functionality in subsequent releases.

### Conclusion

Here, we present MetaRanker, a novel computational pipeline for efficient and accurate assessment of antibiotic resistome risk in metagenomic samples. MetaRanker integrates the abundance and co-occurrence of antibiotic resistance genes (ARGs), mobile genetic elements (MGEs), and virulence factors (VFs) into a unified risk index (RI). By leveraging sequencing reads for precise quantification, along with an optimized database and alignment strategy, the tool achieves high computational efficiency without compromising accuracy. Validation on diverse environmental samples demonstrates MetaRanker’s effectiveness in discriminating resistance risks and quantifying the impact of intervention processes, underscoring its utility in environmental monitoring and risk management. As a robust and scalable solution, MetaRanker is well-suited for profiling antibiotic resistance across various environments. Future development will focus on database refinement and the incorporation of microbial host information to further enhance predictive performance.

## Data Availability

The public sequencing data used in this study are downloaded from Sequence Read Archive (SRA) of National Center for Biotechnology Information (NCBI) or Genome Sequence Archive (GSA) of China National Center for Bioinformation. The accession numbers for all public datasets are listed in Tables S2 to S6. The scripts used for the analysis reported in this study are publicly available at https://github.com/SteamedFish6/MetaRanker-utils. Project name: MetaRanker. Project homepage: https://github.com/SteamedFish6/MetaRanker. Operating system: Linux. Programming language: Python. Other requirements: Python 3.8 or higher with Numpy, Pandas, and Biopython installed, BLASTn, CD-HIT, BWA, Minimap2, SAMtools. License: MIT License.

## SUPPLEMENTAL MATERIAL

Supplemental material aem.02422-25-s0001.pdf Fig. S1 to S3; Tables S1 to S9.

Graphical abstract aem.02422-25-s0002.tif Visual depiction of the study.

ASM does not own the copyrights to Supplemental Material that may be linked to, or accessed through, an article. The authors have granted ASM a non-exclusive, world-wide license to publish the Supplemental Material files. Please contact the corresponding author directly for reuse.
