# Supplementary material for: MetaRanker: precise profiling of antibiotic resistome risk in metagenomes by integrating abundance and genetic co-occurrence
Source: Appl Environ Microbiol. 2026 Feb 18;92(3):e02422-25. doi: 10.1128/aem.02422-25 (PMC12997815; doi:10.1128/aem.02422-25)
Supplement: Supplemental material — Fig. S1 to S3; Tables S1 to S9. [file aem.02422-25-s0001.pdf]

Supplementary materials

MetaRanker: precise profiling of antibiotic resistome risk in metagenomes by integrating abundance and genetic co-occurrence

Zhenyu Guo[1], Yao Xiao[1], Junqiao Zhao[1], Zizhen Tang[2], Yufei Lin[1], Kun Yang[*1]

1. Department of Pharmaceutical & Biological Engineering, School of Chemical Engineering, Sichuan University, Chengdu 610065, China.
2. Key Laboratory of Bio-Resources and Eco-Environment of Ministry of Education, College of Life Sciences, Sichuan University, Chengdu 610065, China.

*Correspondence to:

Kun Yang

cookyoung@scu.edu.cn

Fig. S1. Validation of MetaRanker's abundance calculation and assembly methods. A, B, C: Linear regression and consistency analysis between the risk vector values of MetaRanker and the ARGs, MGEs, and VFs abundance represented by RPM, RPKM, and BPM, respectively; D: Linear regression analysis of RI calculated from contigs assembled using Megahit and MetaSpades; E: Consistency analysis of RI calculated from contigs assembled using Megahit and MetaSpades, represented by the Bland-Altman plot; F: N50 and average length of contigs assembled by Megahit and MetaSpades; G: Number and L50 of contigs assembled by Megahit and MetaSpades; H: Number of REs identified from contigs assembled by Megahit and MetaSpades. This analysis used 48 representative samples (See Table S3. Since MetaSpades requires higher computational resources, only 29 out of the 48 samples used for validation successfully completed assembly).

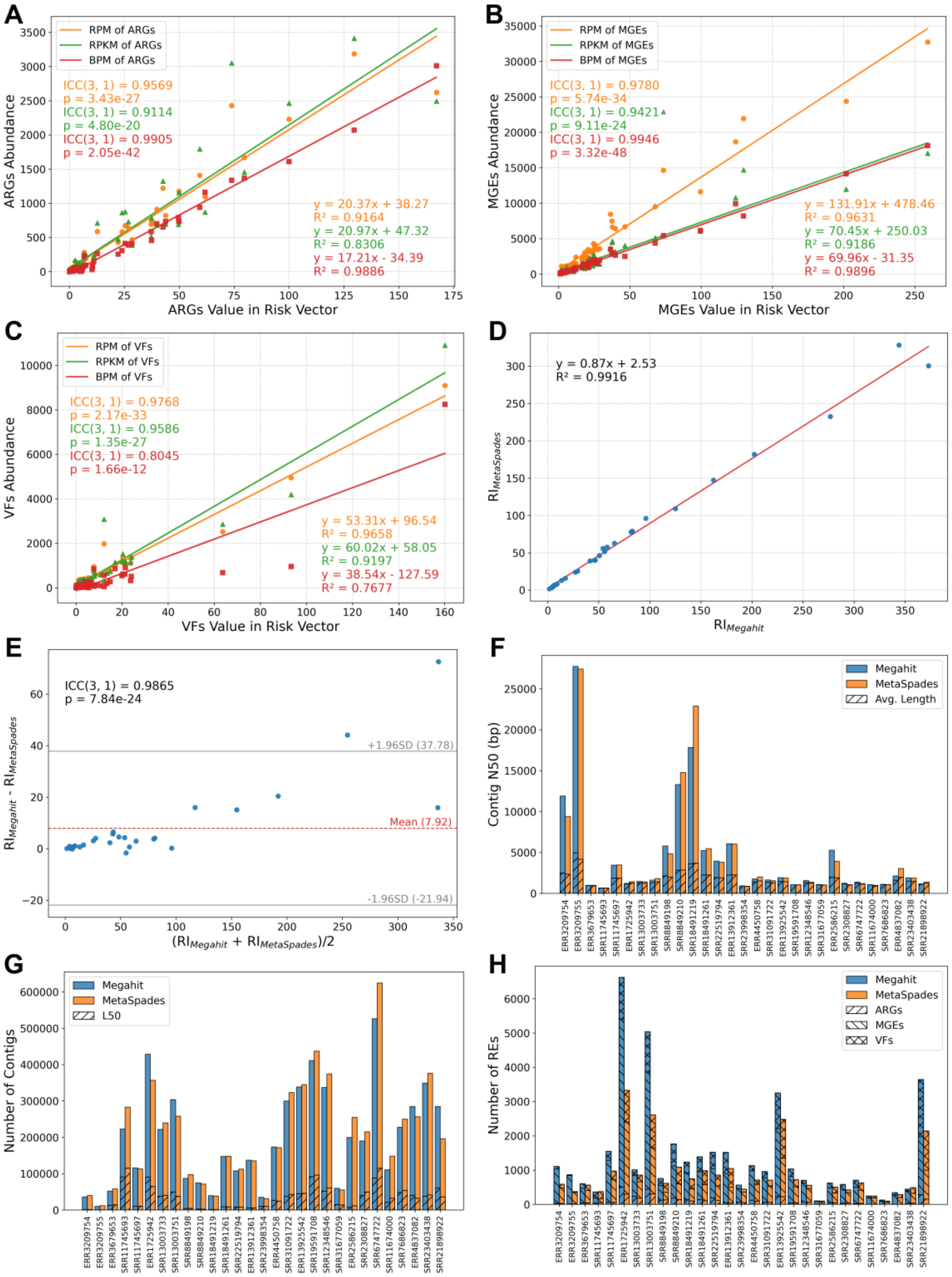
A
RPM of ARGs
RPKM of ARGs
BPM of ARGs
ICC(3, 1) = 0.9569
p = 3.43e-27
ICC(3, 1) = 0.9114
p = 4.80e-20
ICC(3, 1) = 0.9905
p = 2.05e-42
y = 20.37x + 38.27
R² = 0.9164
y = 20.97x + 47.32
R² = 0.8306
y = 17.21x - 34.39
R² = 0.9886
ARGs Abundance
ARGs Value in Risk Vector
B
RPM of MGEs
RPKM of MGEs
BPM of MGEs
ICC(3, 1) = 0.9780
p = 5.74e-34
ICC(3, 1) = 0.9421
p = 9.11e-24
ICC(3, 1) = 0.9946
p = 3.32e-48
y = 131.91x + 478.46
R² = 0.9631
y = 70.45x + 250.03
R² = 0.9186
y = 69.96x - 31.35
R² = 0.9896
MGEs Abundance
MGEs Value in Risk Vector
C
RPM of VFs
RPKM of VFs
BPM of VFs
ICC(3, 1) = 0.9768
p = 2.17e-33
ICC(3, 1) = 0.9586
p = 1.35e-27
ICC(3, 1) = 0.8045
p = 1.66e-12
y = 53.31x + 96.54
R² = 0.9658
y = 60.02x + 58.05
R² = 0.9197
y = 38.54x - 127.59
R² = 0.7677
VFs Abundance
VFs Value in Risk Vector
D
y = 0.87x + 2.53
R² = 0.9916
RI_MetaSpades
RI_Megahit
E
ICC(3, 1) = 0.9865
p = 7.84e-24
+1.96SD (37.78)
Mean (7.92)
-1.96SD (-21.94)
RI_Megahit - RI_MetaSpades
(RI_Megahit + RI_MetaSpades)/2
F
Megahit
MetaSpades
Avg. Length
Contig N50 (bp)
G
Megahit
MetaSpades
L50
Number of Contigs
H
Megahit
MetaSpades
ARGs
MGEs
VFs
Number of REs
ERR3209754
ERR3209755
ERR3679653
SRR11745693
SRR11745697
ERR1725942
SRR13003733
SRR13003751
SRR8849198
SRR8849210
SRR18491219
SRR18491261
SRR22519794
SRR13912361
SRR23998354
ERR4450758
SRR31091722
SRR13925542
SRR19591708
SRR12348546
SRR31677059
ERR2586215
SRR2308827
SRR6747722
SRR11674000
SRR7686823
ERR4837082
SRR23403438
SRR21898922

Fig. S2. Visualization of high risk contigs with multiple REs' co-linearity in hospital sample ERR3209754. Only contigs containing three or more REs are displayed. Red indicates ARGs, green indicates MGEs, and blue indicates VFs (not shown in the figure).

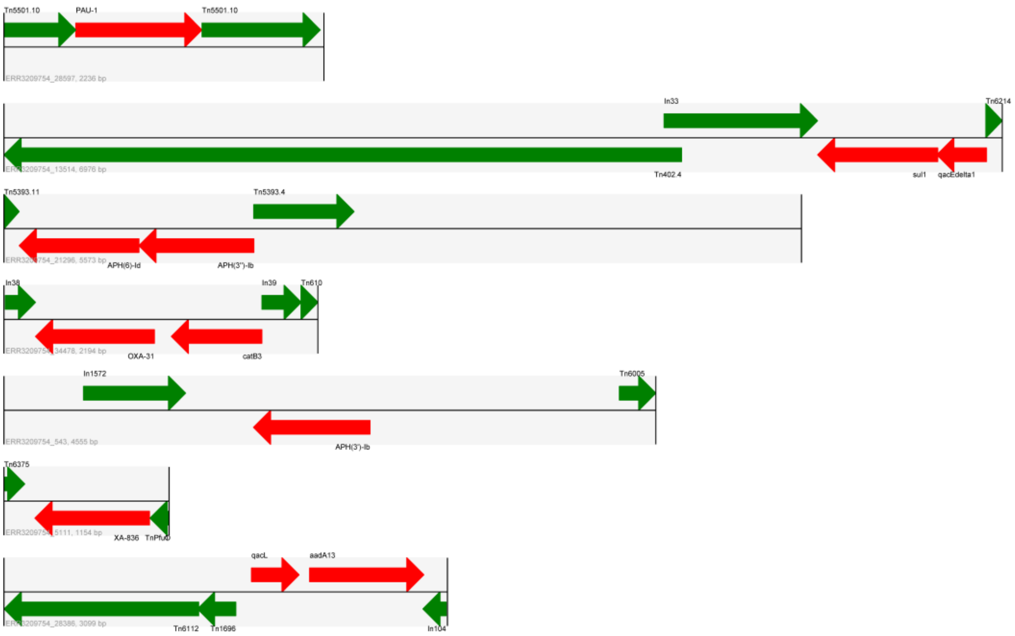

Fig. S3. The distribution of weighted RE abundance ($\bar{d}_{RE} \times w_{RE}$, RE = ARGs, MGEs or VFs) between in-house dataset (n=103) and public dataset (n=240). Independent samples t-test was applied to validate the consistency between these two datasets after scaling by a same group of w_{RE}.

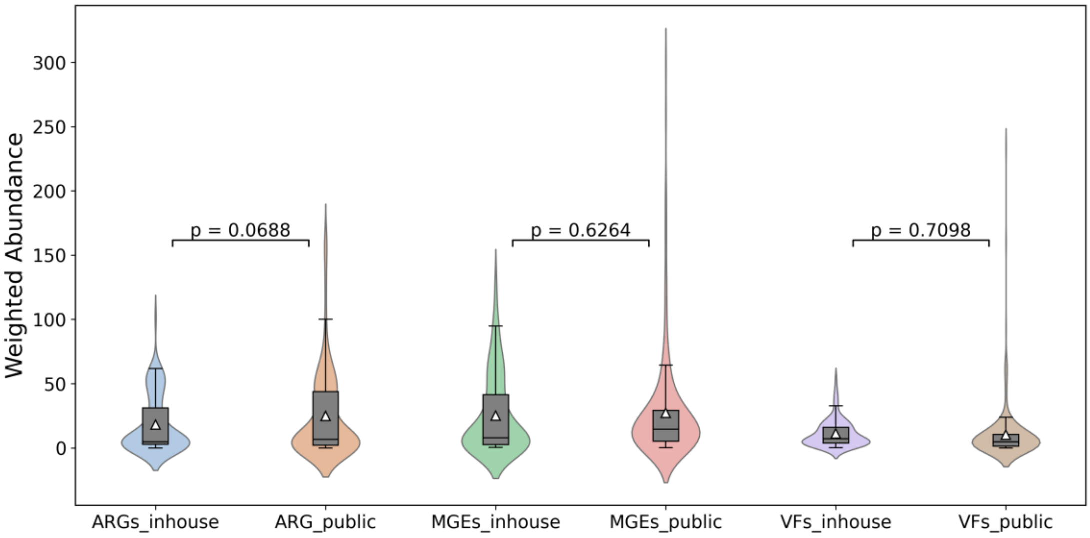

Table S1. Detailed information of reference sequences used for generating reads of mock samples.

Type	Accession ID	Description	Length
the ESKAPE pathogen	CP142124.1	Enterobacter wuhouensis strain AV1 chromosome	4821649
	NC_002516.2	Pseudomonas aeruginosa PAO1	6264404
	NC_004337.2, NC_004851.1	Shigella flexneri 2a str. 301 chromosome, plasmid pCP301	4607202, 221618
	NC_003197.2, NC_003277.2	Salmonella enterica subsp. enterica serovar Typhimurium str. LT2, plasmid pSLT	4857450, 93933
	NC_007795.1	Staphylococcus aureus subsp. aureus NCTC 8325 chromosome	2821361
	NC_016845.1, NC_016838.1, NC_016846.1, NC_016839.1, NC_016840.1, NC_016847.1, NC_016841.1	Klebsiella pneumoniae subsp. pneumoniae HS11286 chromosome, plasmid pKPHS1, pKPHS2, pKPHS3, pKPHS4, pKPHS5 and pKPHS6	5333942, 122799, 111195, 105974, 3751, 3353, 1308
	NC_021870.1, NC_021871.1	Salmonella bongori N268-08 chromosome, plasmid RM1	4683551, 89986
	NZ_CP007470.1	Haemophilus influenzae strain 477 chromosome	1846259
	NZ_CP043318.1, NZ_CP043319.1	Enterobacter chengduensis strain WCHECl-C4 chromosome, plasmid pLAP2_050004	5111427, 106698
	NZ_CP020549.1, NZ_CP020550.1	Streptococcus pneumoniae strain Hu17 chromosome, plasmid pSpn_Hu17	2148350, 6012
	NZ_NIYS01000001.1-NZ_NIYS01000154.1	Shigella boydii strain ESBL-W3-2 contigs	-
	NZ_CP043953.1, NZ_CP043954.1	Acinetobacter baumannii strain K09-14 chromosome, plasmid pK09-14	3972439, 7791
	NZ_CP027986.1	Enterobacter sichuanensis strain SGAir0282 chromosome	4711389
	NZ_CP038996.1, NZ_CP038997.1	Enterococcus faecium strain SRR24 chromosome, plasmid pSRR24	2796178, 123020
	NZ_AP023069.1, NZ_AP023070.1	Neisseria gonorrhoeae strain TUM19854 chromosome, plasmid pMTY19854	2167602, 4153
	NZ_CP055292.1	Shigella sonnei strain SE6-1 chromosome	4762774
	NZ_CP071982.1,	Helicobacter pylori strain MT5135	1615199,

	NZ_CP071983.1	chromosome, plasmid pMT5135	9259
	NZ_JAHZBQ010000001.1-NZ_JAHZBQ010000005.1	MAG: Campylobacter sp. isolate HRGM_Genome_2089 HRGM_Genome_2089_1-5	1067368, 582804, 388907, 101672, 45370
	NZ_CP055055.1, NZ_CP055056.1	Shigella dysenteriae strain SWHEFF_49 chromosome, plasmid unnamed1	5075418, 117256
	NZ_CP141063.1	Enterobacter chuandaensis strain AEC chromosome	4874952
	NZ_CABMME010000002.1, NZ_CABMME010000001.1	Enterobacteriaceae bacterium isolate MGYG-HGUT-02477, plasmid unnamed1	5030517, 53350
multi-drug resistance plasmid group 1	KX528699.1	Escherichia coli plasmid unnamed1	15998
	CP021418.1	Escherichia coli strain ICBEC171S plasmid pICBEC171Smcr	31224
	KY582848.1	Escherichia coli strain WCHEC1604 plasmid pMCR_WCHEC1604-IncX4	31229
	CP027257.1	Escherichia coli strain EC11 plasmid unnamed2	31229
	KX032520.1	Escherichia coli strain Af48 plasmid pAF48	31808
	KX570748.1	Escherichia coli strain ECMY3 plasmid pECMCR-1101	32751
	MG557854.1	Escherichia coli voucher culture pure - one microbial species plasmid PN42	32995
	KY770023.1	Escherichia coli strain ICBEC2AM plasmid pICBEC2AM	33051
	KU761327.1	Klebsiella pneumoniae strain SZ04 plasmid pmcr1_IncX4	33287
	KY012276.1	Escherichia coli strain 18HAE25 plasmid pEc_18HAE25	33287
	MF093645.1	Escherichia coli strain 288328 plasmid pMCR-1.2-IT-Ec	33293
	KX084392.1	Escherichia coli strain B65 plasmid pECJS-B65-33	33298
	MF175190.1	Escherichia coli strain ColR598 plasmid pColR598_1	33299
	MF175185.1	Escherichia coli strain PF52 plasmid pPF52	33300
	CP024149.1	Escherichia coli strain 14EC033 plasmid p14EC033b	33301
	MF978387.1	Escherichia coli strain GDT6F49 plasmid	33301

	pHNGDF49	
KX894453.1	Escherichia coli strain IHIT31346 plasmid pMCR-1-IHIT35346	33303
MF449287.1	Escherichia coli strain T71115 plasmid pIBMC_mcr1	33303
KX236309.1	Klebsiella pneumoniae strain KP-6884 plasmid pMCR1.2-IT	33303
KY964067.1	Escherichia coli strain LV23529 plasmid pLV23529-MCR-1.9	33303
KY565556.1	Escherichia coli strain Mcp0271 plasmid pMcp0271	33303
KY689634.1	Escherichia coli strain 31349 plasmid p31349	33303
LC227558.2	Escherichia coli plasmid pKT2378 DNA	33304
LT838201.1	Escherichia coli isolate WI2 isolate genome assembly	33304
CP018773.2	Escherichia coli strain 2016C-3936C1 plasmid pMCR-1-CT	33304
CP019908.1	Escherichia coli strain MDR_56 plasmid pMCR1-NY	33304
CP015977.1	Escherichia coli strain ICBEC72H plasmid pICBEC72Hmcr	33304
KY770024.1	Escherichia coli strain ICBEC3AM plasmid pICBEC3AM	33304
CP021419.1	Escherichia coli strain ICBEC12-3F plasmid pICBEC12-3mcr	33304
CP028167.1	Escherichia coli strain CFSAN064036 plasmid pGMI17-004_2	33305
KX254343.1	Escherichia coli strain GD-8 plasmid pECGD-8-33	33307
KY120364.1	Salmonella enterica subsp. enterica serovar Typhimurium strain NG14043 plasmid pNG14043	33308
MF175186.1	Escherichia coli strain PF11 plasmid pPF11	33308
MF774188.1	Escherichia coli strain SHP49 plasmid pHNSHP49	33309
KX711706.1	Escherichia coli strain CSZ4 plasmid pCSZ4	33309
KY463454.1	Escherichia coli strain WCHEC1618 plasmid pMCR_WCHEC1618	33309
KY463451.1	Escherichia coli strain WCHEC1606 plasmid pMCR_WCHEC1606	33309
KX772777.1	Escherichia coli strain E15004 plasmid	33309

	pE15004	
MG210937.1	Escherichia coli strain GZ49260 plasmid pGZ49260	33309
MF774184.1	Escherichia coli strain SHP23 plasmid pHNSHP23	33309
MF774182.1	Escherichia coli strain SHP10 plasmid pHNSHP10	33309
MH061196.1	Klebsiella pneumoniae strain AHM7C25I plasmid pHNAH25I-MCR	33310
AP018411.1	Klebsiella pneumoniae plasmid pRYU3223C-1 RYU 3223 DNA	33310
CP028174.1	Salmonella enterica strain CFSAN064033 plasmid pGMI17-001_2	33310
MF175184.1	Escherichia coli strain PF91 plasmid pPF91	33310
KU743383.1	Escherichia coli strain ENV-187 plasmid pESTMCR	33311
KX447768.1	Escherichia coli strain MCR1_NJ plasmid pMCR1-NJ-IncX4	33395
CM007714.1	Escherichia coli strain 3431F plasmid pMCRpoa	33511
MTJV01000045.1	Escherichia coli strain 3431F plasmid pMCRpoa	33511
MF175191.1	Escherichia coli strain CDF8 plasmid pCDF8	33660
MG210939.1	Escherichia coli strain GZ49269 plasmid pGZ49269	33858
CP019072.1	Escherichia coli strain CRE1493 plasmid p1493-1	33858
MG557852.1	Escherichia coli voucher pure culture - one microbial species plasmid PN23	33858
CP024919.1	Klebsiella pneumoniae strain NH54 plasmid pKPNH54.3	33858
CP024041.1	Klebsiella pneumoniae strain QS17-0029 plasmid pMR0617mcr	33858
CP024462.1	Klebsiella pneumoniae strain QS17-0161 plasmid pMR0617mcr1	33858
KX555452.1	Escherichia coli plasmid pMCR-13EC-C962A	34149
KX129783.1	Escherichia coli strain OW3E1 plasmid pOW3E1	34640
KX711708.1	Escherichia coli strain pPY1 plasmid pPY1	34924
KY770025.1	Escherichia coli strain ICBEC13AM	34975

	plasmid pICBEC13AM	
CP017246.1	Escherichia coli strain ICBEC7P plasmid pICBEC7Pmcr	34992
KX711707.1	Escherichia coli strain FS170G plasmid pFS170G	34997
CP024132.1	Escherichia coli strain 14EC007 plasmid p14EC007a	35098
KX555451.1	Escherichia coli plasmid pMCR-11EC-P293	38814
CM008162.1	Klebsiella pneumoniae strain 3111F plasmid pPOAMCR-1KP	38942
NHOE01000122.1	Klebsiella pneumoniae strain 3111F plasmid pPOAMCR-1KP	38942
MG557853.1	Escherichia coli voucher pure culture - one microbial species plasmid PN25	40590
KY120363.1	Salmonella enterica subsp. enterica serovar Typhimurium strain C214 plasmid pC214	42941
CP021078.1	Citrobacter braakii strain SCC4 plasmid pSCC4	44155
KY471146.1	Escherichia coli strain EC111 plasmid pEC111	47038
KX580713.1	Escherichia coli strain ZJ623 plasmid	47409
KY463452.1	Escherichia coli strain WCHEC1622 plasmid pMCR_WCHEC1622	49897
CP024128.1	Escherichia coli strain 14EC001 plasmid p14EC001a	50013
MF990207.1	Escherichia coli strain GDP6F1 plasmid pHNGDF1-1	50435
MF978389.1	Escherichia coli strain GDT6F36 plasmid pHNGDF36-1	52740
MG594800.1	Escherichia coli isolate 1724 plasmid p1724	53926
MG598814.1	Escherichia coli isolate 1670 plasmid p1670	55549
KU922754.1	Kluyvera ascorbata strain WCH1410 plasmid pMCR_1410	57059
MG598816.1	Escherichia coli isolate 979 plasmid p979	58638
MF135536.1	Escherichia coli strain HS20eCTX plasmid pHNHS20EC	59545
KY120366.1	Salmonella enterica subsp. enterica serovar Typhimurium strain R150626 plasmid pR150626	59651
CP021194.1	Escherichia coli strain H17 plasmid	59711

	pH17-1	
MF175187.1	Escherichia coli strain PC11 plasmid pPC11	59830
KX592672.1	Escherichia coli plasmid pEc_04HAE12	59939
KX856067.1	Salmonella enterica subsp. enterica serovar Typhimurium plasmid pHSSH22-MCR1	60035
CP024156.1	Escherichia coli strain 14EC047 plasmid p14EC047a	60258
KY471315.1	Escherichia coli strain M19855 plasmid pMCR-M19855	60357
AP017622.1	Escherichia coli plasmid pMRY15-131_2 DNA	60722
AP018412.1	Escherichia coli plasmid pRYU2912C-1 RYU 2912 DNA	60732
KY363995.1	Shigella sonnei strain SH13Sh069 plasmid pSh069-m6	60733
KY363994.1	Shigella sonnei strain SH12Sh113 plasmid pSh113-m4	60733
KY795977.1	Escherichia coli strain JIE2288 plasmid pJIE2288-1	60733
MG594799.1	Escherichia coli isolate 4222 plasmid p4222	60733
MG598815.1	Escherichia coli isolate 4070 plasmid p4070	60733
KY363998.1	Shigella sonnei strain SH11Sh125 plasmid pSh125-m2	60734
KY471309.1	Escherichia coli strain M15224 plasmid pMCR-M15224	60735
KU934208.1	Escherichia coli strain HeN867 plasmid pHeN867	60757
KX856068.1	Salmonella enterica subsp. enterica serovar Enteritidis plasmid pHSSH23-MCR1	60859
CP022452.1	Salmonella enterica subsp. enterica serovar Indiana strain D90 plasmid pD90-2	60860
MF978388.1	Escherichia coli strain GDT6F93 plasmid pHNGDF93	60863
KY012274.1	Escherichia coli strain 20COE13 plasmid pEc_20COE13	60923
MF175189.1	Escherichia coli strain ColR598 plasmid pColR598_2	60939
CP029184.1	Escherichia coli strain H9Ecoli plasmid	60942

	pMCR-H9	
MF175188.1	Escherichia coli strain ColR644SK1 plasmid pColR644SK1	60952
KY120365.1	Salmonella enterica subsp. enterica serovar Typhimurium strain P111 plasmid pP111	60960
KY795978.1	Escherichia coli strain JIE3685 plasmid pJIE3685-1	60960
CP021176.1	Escherichia coli strain 5CRE51 plasmid p5CRE51-MCR-1	60961
KX032519.1	Escherichia coli strain Af23 plasmid pAF23	61177
KY471308.1	Escherichia coli strain M15049 plasmid pMCR-M15049	61198
MG594798.1	Escherichia coli strain 6383 plasmid p6383	61198
AP017619.1	Escherichia coli plasmid pMRY15-117_2 DNA	61223
KX013538.1	Escherichia coli strain ABC149 plasmid pABC149-MCR-1	61228
MG489944.1	Escherichia coli strain PN16 plasmid unnamed	61304
KY471310.1	Escherichia coli strain M17059 plasmid pMCR-M17059	61531
KY471145.1	Escherichia coli strain EC019 plasmid pEC019	61572
KY471311.1	Escherichia coli strain M19241 plasmid pMCR-M19241	61584
KY471312.1	Escherichia coli strain M19242 plasmid pMCR-M19242	61632
KY471313.1	Escherichia coli strain M19441 plasmid pMCR-M19441	61653
AP017614.1	Escherichia coli plasmid pMRY16-002_4 DNA	61805
MG557851.1	Escherichia coli voucher pure culture-one microbial species plasmid PN21	61805
LT174530.1	Shigella sonnei plasmid pEG430-1	61826
KY446064.1	Escherichia coli strain GD81 plasmid pGD81-1	61922
KY471144.1	Escherichia coli strain EC006 plasmid pEC006	62174
KY624633.1	Citrobacter braakii strain CA-26 plasmid pCA-26	62214
KU870627.1	Escherichia coli strain VT55363 plasmid	62219

		pVT553	
	NSBS01000056.1	Escherichia coli strain ECO3347 plasmid pMCR-1	62343
	CM008278.1	Escherichia coli strain ECO3347 plasmid pMCR-1	62343
	AP018110.1	Escherichia coli plasmid pMTY17668-MCR1.5 DNA	62375
	KX580716.1	Escherichia coli strain ZJ1635 plasmid	62440
	CP024148.1	Escherichia coli strain 14EC033 plasmid p14EC033a	62585
	KX013539.1	Escherichia coli strain BA77 plasmid pBA77-MCR-1	62661
	MG210940.1	Escherichia coli strain GZ49273 plasmid pGZ49273	62701
	MF693349.1	Escherichia coli strain 3216 plasmid pG3216	62717
	KY405001.1	Escherichia coli strain Ec28 plasmid pEC28	63087
	KY471314.1	Escherichia coli strain M19736 plasmid pMCR-M19736	63230
	CP016405.1	Escherichia coli strain 210221272 plasmid pSLy21	63329
	KY853650.2	Escherichia coli strain 347-43491A plasmid p977565	63381
	CP021205.1	Escherichia coli strain Z1002 plasmid p1002-MCR1	63392
	KY363996.1	Shigella sonnei strain SH11Sh487 plasmid pSh487-m4	63512
	MG725031.1	Escherichia coli strain SDX5C133 plasmid pHNSD133-MCR	63568
	KX254342.1	Escherichia coli strain JS-61 plasmid pECJS-61-63	63656
	KX084393.1	Escherichia coli strain 61 plasmid pECJS-61-63	63656
	KY693674.1	Escherichia coli strain OM97 plasmid pOM97-mcr	63722
	KY792081.1	Escherichia coli strain EC-MCR1.8 plasmid unnamed	63839
	CP024135.1	Escherichia coli strain 14EC017 plasmid p14EC017a	63978
	KP347127.1	Escherichia coli strain SHP45 plasmid pHNSHP45	64015
	KY363997.1	Shigella sonnei strain SH11Sh418 plasmid pSh418-m3	64266

	KY363999.1	Shigella sonnei strain SH10Sh016 plasmid pSh016-m1	64266
	CP018106.1	Escherichia coli strain MRSN352231 plasmid pMR0716_mcr1	64467
	CP018118.1	Escherichia coli strain MRSN346638 plasmid pMRSN346638_64.5	64467
	CP018112.1	Escherichia coli strain MRSN346595 plasmid pMRSN346595_64.5	64467
	KY471307.1	Escherichia coli strain GN775 plasmid pMCR-GN775	64600
	KY565557.1	Escherichia coli strain Mcp0221 plasmid pMcp0221	64664
	CP024139.1	Escherichia coli strain 14EC020 plasmid p14EC020a	64765
	KU761326.1	Escherichia coli strain SZ02 plasmid pmcr1_IncI2	64964
	KX505142.1	Cronobacter sakazakii strain WF-5-19C plasmid pWF-5-19C_mcr-1	65203
	CP019052.1	Escherichia coli strain CRE1540 plasmid p1540-1	65533
	CP018124.1	Escherichia coli strain MRSN346355 plasmid pMRSN346355_65.5	65539
	CP015913.1	Escherichia coli strain 210205630 plasmid pSLy1	65888
	MG210938.1	Escherichia coli strain GZ49266 plasmid pGZ49266	66183
	CP024142.1	Escherichia coli strain 14EC029 plasmid p14EC029a	66596
	KX034083.1	Escherichia coli strain A31-12 plasmid pA31-12	67134
	CP025679.1	Escherichia albertii strain ChinaSP140150 plasmid pEA-3	68747
	MF510496.1	Klebsiella pneumoniae strain SCKP-LL83 plasmid pMCR_SCKP-LL83	97393
	KX518745.1	Escherichia coli strain HYEC7 plasmid pHYEC7-mcr1	97559
	KX129784.1	Escherichia coli strain H226B plasmid pH226B	209401
multi-drug resistance plasmid group 2	MF136779.1	Escherichia coli strain HKSHmcr1_P2_EC plasmid pHKSHmcr1_P2_p2	33309
	MF381176.1	Escherichia coli strain V163 plasmid pV163M	36502
	MF136778.1	Escherichia coli strain	47818

	HKSHmcr1_P2_EC plasmid pHKSHmcr1_P2_p1	
CP016550.1	Escherichia coli strain O177:H21 plasmid unnamed4	49695
KX377410.1	Klebsiella pneumoniae strain WCHKP1511 plasmid pMCR_1511	57278
MG552133.1	Escherichia coli strain ECSC102 plasmid pECSC102	60744
KX013540.1	Escherichia coli strain BA76 plasmid pBA76-MCR-1	64942
KY012275.1	Escherichia coli strain 27COE18 plasmid pEc_27COE18	65216
KX772778.1	Escherichia coli strain E15017_00 plasmid pE15017_00	65375
KU934209.1	Salmonella enterica strain SC23 plasmid pSCS23	65419
CP019052.1	Escherichia coli strain CRE1540 plasmid p1540-1	65533
MH213346.1	Escherichia coli strain EC1188 plasmid pEC1188-MCR	65806
KY802014.1	Escherichia coli strain ZE36 plasmid pZE36	65846
MG515249.1	Escherichia coli strain EC16-50 plasmid pEC16-50-MCR	65978
KX034083.1	Escherichia coli strain A31-12 plasmid pA31-12	67134
KU353730.1	Escherichia coli plasmid pKH457-3-BE	79798
KX443408.2	Klebsiella pneumoniae strain SC24 plasmid pKSC24	89790
KU994859.1	Escherichia coli strain KP81 plasmid pKP81-BE	91041
KY689635.1	Escherichia coli strain Mbl536 plasmid pMbl536	100230
KY565558.1	Escherichia coli strain Mbl488 plasmid pMbl488	101065
CP022169.1	Salmonella enterica subsp. enterica serovar Typhimurium strain WW012 plasmid pWW012	151609
MG591702.1	Escherichia coli strain EC36 plasmid pMCR-EC36	164803
CP029493.1	Escherichia coli strain HS30-1 plasmid pHS30-1	179444
KX856065.1	Salmonella enterica subsp. enterica serovar Typhimurium plasmid pASSD2-	187257

	MCR1	
NKYM01000303.1	Escherichia coli strain LH57 plasmid pLH57-mcr1	218800
CM008264.1	Escherichia coli strain LH57 plasmid pLH57-mcr1	218800
MF678350.1	Escherichia coli strain WCHEC-LL123 plasmid pMCR1_WCHEC-LL123	223698
CM008265.1	Escherichia coli strain LH30 plasmid pLH30-mcr1	223898
NKYL01000255.1	Escherichia coli strain LH30 plasmid pLH30-mcr1	223898
CP021209.1	Escherichia coli strain strain Z247 plasmid p2474-MCR1	223982
MH208235.1	Escherichia coli strain APECA2 plasmid pJMA2	224630
KX276657.1	Escherichia coli strain MRSN388634 plasmid pMR0516mcr	225069
MG662415.1	Escherichia coli strain EC1107 plasmid EC1107-IncHI2-226K	226105
CP023143.1	Escherichia coli strain CFSAN061770 plasmid pEGY1-MCR-1	228947
MH128771.1	Escherichia coli strain 803DBmcr plasmid 803-DB-mcr	230365
KY689632.1	Escherichia coli strain 19-M12 plasmid p19M12	232345
CP022165.1	Escherichia coli strain M160133 plasmid pM160133_p1	233149
KX023262.1	Escherichia coli plasmid pSCE516-1	237939
KX856066.1	Salmonella Typhimurium strain HSHLJ1 plasmid pHSHLJ1-MCR1	238539
CM008266.1	Escherichia coli strain 1rc4 plasmid p1rc4-mcr1	239098
NKYK01000096.1	Escherichia coli strain 1rc4 plasmid p1rc4-mcr1	239098
KU743384.1	Escherichia coli strain SA26 plasmid pSA26-MCR-1	240367
CP025402.1	Escherichia coli strain MS8345 plasmid pMS8345A	241162
KX084394.1	Escherichia coli strain 59 plasmid pECJS-59-244	243572
KX129782.1	Escherichia coli strain S38 plasmid pS38	247885
NKYJ01000136.1	Escherichia coli strain LH1 plasmid pLH1-mcr1	248198
CM008267.1	Escherichia coli strain LH1 plasmid	248198

		pLH1-mcr1	
	KU341381.1	Escherichia coli strain SHP45 plasmid pHNSHP45-2	251493
	MF135535.1	Raoultella ornithinolytica strain TS53CTX plasmid pHNTS53-1	253021
	MF135534.1	Raoultella ornithinolytica strain TS48CTX plasmid pHNTS48-1	253021
	KY990887.1	Escherichia coli strain XG-E1 plasmid pXGE1mcr	254048
	KY689633.1	Escherichia coli strain 100R plasmid p100R	256260
	CP026492.1	Escherichia coli strain HS13-1 plasmid pHS13-1-IncHI2	264344
	CP020493.1	Salmonella enterica subsp. enterica strain 08-00436 plasmid pSE08-00436-1	264914
	CP027202.1	Escherichia coli strain WCHEC025943 plasmid pMCR1_025943	265538
	KX254341.1	Escherichia coli strain JS-B60 plasmid pECJS-B60-267	267486
	CP029748.1	Escherichia coli strain 2016C-3878 plasmid pMCR1-PA	276880
	CP019214.2	Escherichia coli strain WCHEC050613 plasmid pMCR_WCHEC050613	289112
	MG656414.1	Escherichia coli strain 15-50 plasmid pEC15-MCR-50	350179
	MG591698.1	Escherichia coli strain PN43 plasmid unnamed	353691
	CP017632.1	Escherichia coli SLK172 plasmid pSLK172-1	369298
common environmental bacteria genome	NC_013739.1	Conexibacter woesei DSM 14684	6359369
	NZ_HE997181.1	Reyranella massiliensis 521	5792259
	NZ_AP014648.1	Methyloceanibacter caenitepidi strain Gela4 chromosome	3424964
	NZ_CP015136.1	Luteitalea pratensis strain DSM 100886 isolate HEG_-6_39 chromosome	7480314
	NZ_CP110813.1	Lysobacter enzymogenes strain B25 chromosome	6358104
	NZ_CP029788.1	Streptomyces actuosus strain ATCC 25421 chromosome	8145579
	NZ_CP031417.1	Pseudolabrys taiwanensis strain CC-BB4 isolate CCUG 51779 chromosome	5593741
	NZ_CP036271.1	Caulifigura coniformis strain Pan44 chromosome	6761146
	NZ_CP036274.1	Anatilimnocola aggregata strain ETA_A8	9007740

		chromosome	
	NZ_CP042430.1	Baekduia soli strain BR7-21 chromosome	5212251
	NZ_CP042906.1	Hypericibacter terrae strain R5913 chromosome	5894118
	NZ_JAFBCT010000001.1	Agromyces cerinus strain VKM Ac-1351 Ga0451117_01	3977730
	NZ_CP087164.1	Capillimicrobium parvum strain 0166_1 chromosome	5953270
	NZ_CP113162.1	Devosia riboflavina strain IFO13584 chromosome	3271440
	NZ_BSVA01000001.1	Homoserinibacter gongjuensis strain NBRC 108755 sequence1	3290533
	NZ_CP012700.1, NZ_CP012701.1, NZ_CP012702.1, NZ_CP012703.1, NZ_CP012704.1, NZ_CP012705.1	Sphingopyxis macrogoltabida strain EY-1 chromosome, isolate activated sludge plasmid 1-5	4757879, 196952, 58892, 34474, 30496, 19860
	NZ_CP015880.1, NZ_CP015881.1, NZ_CP015882.1	Ensifer adhaerens strain Casida A chromosome, plasmid pCasidaAA and plasmid pCasidaAB	4071185, 1736943, 1459374
	NZ_PZZZ01000001.1-NZ_PZZZ01000022.1	Mycoplana dimorpha strain DSM 7138 Ga0215684_101-122	-

Table S2. Detailed information of the 9 samples used for sequencing depth benchmarking, downloaded from the SRA public database.

Sample	Environment	Risk Vector			Risk Modulus	Co-occur Score	Risk Index	Number of Reads	Number of Bases	Number of Contigs
		ARGs	MGE	VFs						
SRR18491219	human feces	61.768	24.525	16.961	68.589	1.401	96.118	3.91E+07	5.85E+09	3.94E+04
SRR22519794	human feces	167.240	73.646	160.135	242.974	1.416	343.968	3.32E+07	4.98E+09	1.07E+05
SRR26803261	human feces	79.676	46.607	6.658	92.546	1.359	125.757	4.48E+07	6.72E+09	9.47E+04
ERR13925542	municipal sewage	28.477	37.745	21.228	51.830	1.263	65.447	6.70E+07	1.00E+10	3.38E+05
SRR23998354	municipal sewage	12.744	36.479	12.192	40.519	1.149	46.543	8.02E+07	5.90E+09	3.49E+04
SRR31677053	municipal sewage	10.273	18.466	93.347	95.709	1.147	109.734	8.68E+07	1.25E+10	1.56E+05
SRR23403438	soil	1.250	5.111	1.727	5.538	1.088	6.023	4.98E+07	7.47E+09	3.49E+05
ERR4837146	soil	1.423	1.074	2.168	2.807	1.034	2.901	6.80E+07	9.56E+09	3.26E+05
SRR1049280	lake fresh water (QLFW)	0.884	1.257	1.607	2.224	1.000	2.224	6.80E+07	1.02E+10	4.41E+05

Table S3. Detailed information of the 48 samples used for validation, downloaded from the SRA public database.

Sample	Environment	Risk Vector			Risk Modulus	Co-occur Score	Risk Index	Number of Reads	Number of Bases	Number of Contigs
		ARGs	MGE	VFs						
ERR3209754	hospital	59.370	201.787	8.328	210.505	1.314	276.677	2.89E+07	2.89E+09	3.60E+04
ERR3209755	hospital	73.939	258.882	3.172	269.252	1.385	372.977	2.64E+07	2.64E+09	9.80E+03
ERR3674565	hospital	2.070	3.654	3.988	5.792	1.213	7.022	5.79E+07	8.61E+09	7.07E+05
ERR3679653	hospital	6.690	37.449	7.775	38.828	1.067	41.430	1.05E+07	1.44E+09	5.22E+04
SRR26638149	hospital	11.090	19.136	1.266	22.153	1.271	28.149	7.37E+07	1.08E+10	5.59E+05
SRR11745693	hospital	1.981	2.616	3.918	5.111	1.273	6.504	3.79E+07	5.10E+09	2.23E+05
SRR11745697	hospital	23.944	99.568	14.876	103.481	1.209	125.101	4.19E+07	5.50E+09	1.16E+05
ERR1725942	hospital	37.431	129.810	20.004	136.572	1.188	162.292	6.96E+07	1.05E+10	4.29E+05
SRR13003733	hospital	42.588	17.139	1.518	45.932	1.214	55.771	3.54E+07	5.30E+09	2.22E+05
SRR13003751	hospital	100.083	124.250	20.357	160.839	1.257	202.233	4.61E+07	6.91E+09	3.04E+05
SRR8849198	human feces	37.249	25.584	2.068	45.236	1.194	54.012	2.38E+07	5.95E+09	8.67E+04
SRR8849210	human feces	49.646	17.846	21.412	56.935	1.455	82.867	6.66E+07	9.98E+09	7.48E+04
SRR18491219	human feces	61.768	24.525	16.961	68.589	1.401	96.118	3.91E+07	5.85E+09	3.94E+04
SRR18491261	human feces	43.798	10.934	5.635	45.492	1.278	58.155	5.30E+07	7.94E+09	1.47E+05
SRR22519794	human feces	167.240	73.646	160.135	242.974	1.416	343.968	3.32E+07	4.98E+09	1.07E+05
SRR26803261	human feces	79.676	46.607	6.658	92.546	1.359	125.757	4.48E+07	6.72E+09	9.47E+04
SRR26803334	human feces	39.780	23.858	0.114	46.386	1.128	52.330	4.49E+07	6.73E+09	7.30E+04
SRR11845624	livestock feces	49.936	6.599	0.068	50.370	1.252	63.054	7.86E+07	1.18E+10	5.37E+05
SRR11845631	livestock feces	129.696	39.836	1.778	135.688	1.196	162.245	8.93E+07	1.34E+10	4.89E+05
ERR13912361	municipal sewage	10.513	20.472	63.655	67.687	1.206	81.645	8.05E+07	1.20E+10	1.37E+05
SRR8208343	municipal sewage	22.213	67.747	23.687	75.127	1.156	86.844	1.18E+08	1.77E+10	9.26E+05
SRR31677053	municipal sewage	10.273	18.466	93.347	95.709	1.147	109.734	8.68E+07	1.25E+10	1.56E+05
SRR23998354	municipal sewage	12.744	36.479	12.192	40.519	1.149	46.543	8.02E+07	5.90E+09	3.49E+04
ERR4450758	municipal sewage	25.361	28.793	4.116	38.590	1.186	45.765	6.09E+07	6.08E+09	1.73E+05
SRR31091722	municipal sewage	4.362	25.611	3.525	26.218	1.122	29.410	5.62E+07	8.47E+09	3.00E+05
ERR13925542	municipal sewage	28.477	37.745	21.228	51.830	1.263	65.447	6.70E+07	1.00E+10	3.38E+05

SRR8206192	municipal sewage	6.428	16.355	13.433	22.119	1.113	24.629	8.77E+07	1.31E+10	5.04E+05
SRR19591708	treated sewage	3.047	23.956	5.133	24.688	1.092	26.954	5.62E+07	8.49E+09	4.11E+05
SRR12348546	treated sewage	2.709	8.172	1.207	8.693	1.072	9.319	5.95E+07	8.92E+09	3.37E+05
DRR438475	treated sewage	4.735	25.796	12.270	28.956	1.101	31.867	2.25E+08	3.37E+10	6.91E+05
DRR438511	treated sewage	1.620	27.561	6.195	28.295	1.141	32.279	2.49E+08	3.74E+10	2.37E+05
SRR31677059	treated sewage	1.017	1.125	0.054	1.517	1.074	1.629	6.78E+07	9.58E+09	5.99E+04
ERR2586215	treated sewage	7.517	12.260	2.813	14.654	1.178	17.258	5.38E+07	6.77E+09	2.00E+05
SRR14120362	treated sewage	0.713	3.743	0.295	3.822	1.099	4.201	5.36E+07	7.76E+09	2.95E+05
SRR14120376	treated sewage	3.468	20.758	4.217	21.464	1.111	23.839	6.03E+07	8.73E+09	3.28E+05
SRR2308827	activated sludge	2.576	12.083	1.805	12.485	1.083	13.526	5.06E+07	5.10E+09	1.90E+05
SRR6747722	activated sludge	0.654	5.943	3.529	6.943	1.078	7.486	4.95E+07	7.41E+09	5.26E+05
SRR11674000	activated sludge	0.168	4.020	1.867	4.435	1.062	4.709	3.03E+07	4.51E+09	1.10E+05
SRR11674002	activated sludge	2.423	10.509	5.641	12.171	1.053	12.820	1.93E+08	2.85E+10	1.31E+06
SRR8205411	activated sludge	3.119	9.360	4.868	11.002	1.090	11.989	1.05E+08	1.58E+10	7.98E+05
SRR7686823	wilderness river water	0.104	5.865	0.403	5.880	1.171	6.888	2.59E+07	3.88E+09	2.28E+05
SRR21941333	urban river water	5.905	8.832	10.542	14.967	1.079	16.147	1.11E+08	1.57E+10	5.14E+05
ERR4837082	soil	0.018	4.158	0.599	4.201	1.033	4.340	6.23E+07	7.19E+09	2.84E+05
ERR4837146	soil	1.423	2.168	1.074	2.807	1.034	2.901	6.80E+07	9.56E+09	3.26E+05
SRR17642938	soil	5.431	1.137	5.540	7.841	1.021	8.003	9.23E+07	1.38E+10	5.03E+05
SRR18494403	soil	4.635	1.532	8.007	9.378	1.046	9.811	1.15E+08	1.74E+10	6.43E+05
SRR23403438	soil	1.250	5.111	1.727	5.538	1.088	6.023	4.98E+07	7.47E+09	3.49E+05
SRR21898922	bird feces	26.789	21.311	23.998	41.806	1.211	50.612	8.61E+07	1.29E+10	2.85E+05

Table S4. Information of 103 metagenomic samples from our previous studies. For accession ID starts with “CRR”, detailed descriptions can be found at Genome Sequence Archive of China National Center for Bioinformation (https://ngdc.cncb.ac.cn/gsa/). For accession ID starts with “SRR”, detailed descriptions can be found at NCBI (https://www.ncbi.nlm.nih.gov/).

Environment	Sample	Accession ID	Meta Information	Risk Vector			Risk Modulus	Cooccur Score	Risk Index	Number of Reads	Number of Bases	Number of Contigs	Mean of Risk Index	SD of Risk Index
				ARGs	MGE	VFs								
farmland (n=41)	EP.W1A	CRR1673733	Sludge amended farmland soil in Chengdu, Sichuan Province(1), EP=eggplant, MZ=maize, SF=sunflower, OS=original soil, SAS=sludge amended soil, and W means week	4.583	2.178	4.144	6.552	1.044	6.839	4.05E+07	6.07E+09	1.41E+05	8.685	3.761
	EP.W1B	CRR1673734		2.516	4.294	3.169	5.900	1.068	6.304	5.95E+07	8.91E+09	2.38E+05		
	EP.W4A	CRR1673735		3.820	2.687	11.369	12.291	1.046	12.852	4.04E+07	6.05E+09	1.60E+05		
	EP.W4B	CRR1673736		3.161	4.018	3.426	6.154	1.067	6.566	5.30E+07	7.93E+09	2.16E+05		
	EP.W4C	CRR1673737		3.804	5.532	7.257	9.886	1.056	10.437	5.01E+07	7.46E+09	2.10E+05		
	EP.W6A	CRR1673738		5.866	4.611	22.087	23.313	1.066	24.857	4.30E+07	6.45E+09	2.28E+05		
	EP.W6B	CRR1673739		4.404	2.108	6.349	8.009	1.059	8.482	5.58E+07	8.36E+09	1.96E+05		
	EP.W6C	CRR1673740		4.633	2.533	7.065	8.820	1.053	9.286	4.49E+07	6.72E+09	1.73E+05		
	EP.W14A	CRR1673741		3.106	1.673	5.147	6.240	1.022	6.375	4.56E+07	6.84E+09	1.39E+05		
	EP.W14B	CRR1673742		2.482	1.854	5.843	6.613	1.057	6.988	4.90E+07	7.34E+09	1.97E+05		
	EP.W14C	CRR1673743		5.202	3.986	12.047	13.714	1.052	14.431	5.54E+07	8.29E+09	3.85E+05		
	MZ.W1A	CRR1673744		2.104	3.887	3.222	5.469	1.058	5.786	4.71E+07	7.05E+09	2.31E+05		
	MZ.W1B	CRR1673745		2.871	5.144	2.514	6.405	1.081	6.924	5.49E+07	8.22E+09	3.27E+05		
	MZ.W1C	CRR1673746		1.746	2.569	2.280	3.853	1.077	4.151	6.08E+07	9.10E+09	1.96E+05		
	MZ.W4A	CRR1673747		3.672	3.020	6.216	7.825	1.095	8.567	4.35E+07	6.52E+09	1.58E+05		
	MZ.W4B	CRR1673748		3.475	3.366	7.224	8.694	1.075	9.348	5.38E+07	8.06E+09	2.61E+05		
	MZ.W4C	CRR1673749		3.086	4.201	4.322	6.771	1.073	7.267	4.70E+07	7.05E+09	2.31E+05		
	MZ.W6A	CRR1673750		4.088	3.211	6.210	8.099	1.035	8.385	4.49E+07	6.74E+09	1.69E+05		
	MZ.W6B	CRR1673751		2.494	1.773	7.010	7.649	1.048	8.020	5.34E+07	8.00E+09	1.56E+05		
	MZ.W6C	CRR1673752		3.765	2.793	4.843	6.740	1.041	7.014	4.56E+07	6.83E+09	1.56E+05		
	MZ.W14A	CRR1673753		5.541	3.378	15.788	17.070	1.041	17.762	4.22E+07	6.33E+09	2.39E+05		
	MZ.W14B	CRR1673754		2.480	1.869	5.450	6.272	1.038	6.514	4.52E+07	6.78E+09	1.85E+05		
	MZ.W14C	CRR1673755		2.762	1.921	5.933	6.820	1.036	7.068	4.56E+07	6.83E+09	1.83E+05		
	SF.W1A	CRR1673765		3.820	2.917	4.151	6.351	1.063	6.754	4.31E+07	6.46E+09	1.83E+05		

	SF.W1B	CRR1673766		2.667	4.412	5.479	7.523	1.113	8.371	4.23E+07	6.34E+09	2.25E+05		
	SF.W1C	CRR1673767		2.791	6.724	2.282	7.630	1.078	8.229	4.67E+07	7.00E+09	3.58E+05		
	SF.W4A	CRR1673756		4.365	2.426	6.158	7.928	1.053	8.351	4.23E+07	6.34E+09	1.52E+05		
	SF.W4B	CRR1673757		4.660	4.083	7.437	9.679	1.058	10.244	4.53E+07	6.80E+09	2.32E+05		
	SF.W4C	CRR1673758		2.931	2.366	3.639	5.237	1.033	5.408	4.22E+07	6.32E+09	1.23E+05		
	SF.W6A	CRR1673759		2.299	2.062	5.243	6.085	1.042	6.340	4.45E+07	6.67E+09	1.57E+05		
	SF.W6B	CRR1673760		3.822	4.995	5.213	8.169	1.069	8.731	4.40E+07	6.59E+09	2.43E+05		
	SF.W6C	CRR1673761		4.390	3.580	6.371	8.525	1.053	8.980	4.96E+07	7.44E+09	2.45E+05		
	SF.W14A	CRR1673762		5.906	2.580	12.665	14.211	1.041	14.794	5.10E+07	7.65E+09	2.64E+05		
	SF.W14B	CRR1673763		2.636	1.975	4.614	5.669	1.028	5.825	4.44E+07	6.65E+09	2.06E+05		
	SF.W14C	CRR1673764		4.541	2.005	7.187	8.735	1.030	9.000	5.15E+07	7.72E+09	2.16E+05		
	OS.1	CRR1673730		2.800	1.353	2.518	4.002	1.023	4.093	8.19E+07	1.23E+10	1.98E+05		
	OS.2	CRR1673731		2.215	1.260	3.895	4.655	1.080	5.027	8.53E+07	1.28E+10	2.38E+05		
	OS.3	CRR1673732		3.262	1.403	4.559	5.779	1.036	5.985	8.14E+07	1.22E+10	2.40E+05		
	SAS.1	CRR1673727		4.578	7.915	2.902	9.593	1.081	10.375	7.95E+07	1.19E+10	6.47E+05		
	SAS.2	CRR1673728		3.637	7.800	2.349	8.921	1.086	9.692	8.11E+07	1.22E+10	6.31E+05		
	SAS.3	CRR1673729		3.635	7.899	2.206	8.970	1.078	9.666	8.21E+07	1.23E+10	6.65E+05		
urban animal feces (n=7)	JRBE2b	CRR355451	Bird feces collected from Jinjiang River (JR)[1], Yalujiang River (YR) and Yancheng, Jiangsu Province (JY)	101.419	75.492	23.157	128.535	1.167	150.060	4.50E+07	6.75E+09	2.32E+05	61.685	38.619
	JRBE3	CRR355452		17.500	6.733	53.855	57.026	1.193	68.037	4.49E+07	6.72E+09	3.42E+05		
	JRBE4	CRR355453		30.727	19.352	22.753	42.853	1.243	53.247	5.84E+07	8.75E+09	2.10E+05		
	YRBE1	CRR2042948		9.328	24.430	1.121	26.174	1.139	29.816	5.92E+07	8.88E+09	6.18E+04		
	YRBE4	CRR2042951		1.318	31.730	0.397	31.760	1.122	35.633	4.60E+07	6.89E+09	4.45E+04		
	JYBE1	CRR2042952		2.291	5.869	45.808	46.239	1.341	62.016	3.97E+07	5.96E+09	1.07E+05		
	JYBE3	CRR2042953		8.170	10.557	21.575	25.371	1.300	32.987	4.03E+07	6.04E+09	1.06E+05		
urban environment (n=39)	JRW2b	CRR355454	Water samples of Jinjiang River (JR)(2), and municipal wastewater samples	5.153	17.882	2.398	18.763	1.103	20.696	4.12E+07	6.18E+09	3.97E+05	81.006	39.520
	JRW3a	CRR355455		11.715	41.787	9.026	44.327	1.119	49.606	4.09E+07	6.13E+09	3.57E+05		
	JRW4	CRR355456		12.418	37.112	7.551	39.856	1.119	44.589	4.30E+07	6.45E+09	3.74E+05		
	P4.01	CRR2039273		43.718	87.029	22.859	100.039	1.173	117.362	9.76E+07	1.46E+10	7.35E+05		
	P4.02	CRR2039274		30.835	36.349	24.072	53.399	1.149	61.369	9.88E+07	1.48E+10	8.35E+05		

P4.03	CRR2039275	collected from WWTP influent in Chengdu, Sichuan Province, P4=Plant No.4, P7=Plant No.7, P9=Plant No.9	27.408	40.933	9.545	50.178	1.146	57.525	9.15E+07	1.37E+10	8.55E+05
P4.04	CRR2039276		41.444	50.381	10.957	66.150	1.156	76.461	1.17E+08	1.75E+10	9.92E+05
P4.05	CRR2039277		39.006	67.853	19.040	80.549	1.149	92.545	1.08E+08	1.62E+10	9.28E+05
P4.06	CRR2039278		24.151	32.364	16.327	43.558	1.145	49.872	9.06E+07	1.36E+10	7.52E+05
P4.07	CRR2039279		40.928	52.705	32.854	74.379	1.161	86.376	8.93E+07	1.34E+10	7.29E+05
P4.08	CRR2039280		37.491	51.645	18.642	66.486	1.161	77.206	9.52E+07	1.42E+10	7.19E+05
P4.09	CRR2039281		42.751	57.878	14.169	73.336	1.172	85.926	8.10E+07	1.21E+10	5.70E+05
P4.10	CRR2039282		58.875	67.179	35.052	95.958	1.183	113.472	8.07E+07	1.21E+10	6.11E+05
P4.11	CRR2039283		48.987	87.758	22.173	102.921	1.159	119.239	9.92E+07	1.49E+10	7.34E+05
P4.12	CRR2039284		54.038	130.354	22.182	142.843	1.179	168.418	8.80E+07	1.32E+10	6.66E+05
P7.01	CRR2039285		51.073	93.323	14.089	107.313	1.176	126.246	8.25E+07	1.23E+10	6.16E+05
P7.02	CRR2039286		23.843	33.858	5.275	41.746	1.143	47.697	8.23E+07	1.23E+10	7.70E+05
P7.03	CRR2039287		8.853	15.533	2.516	18.055	1.108	19.996	1.00E+08	1.50E+10	8.92E+05
P7.04	CRR2039288		15.942	20.731	4.191	26.485	1.139	30.172	1.08E+08	1.61E+10	1.01E+06
P7.05	CRR2039289		6.282	11.865	2.778	13.709	1.104	15.138	9.00E+07	1.35E+10	7.77E+05
P7.06	CRR2039290		6.646	12.800	2.876	14.707	1.111	16.342	8.48E+07	1.27E+10	8.09E+05
P7.07	CRR2039291		13.847	14.641	5.425	20.869	1.163	24.276	8.51E+07	1.27E+10	8.04E+05
P7.08	CRR2039292		31.441	35.055	11.220	48.408	1.176	56.923	9.24E+07	1.38E+10	8.43E+05
P7.09	CRR2039293		21.483	24.804	6.953	33.542	1.176	39.446	8.28E+07	1.24E+10	7.40E+05
P7.10	CRR2039294		25.085	27.287	9.072	38.159	1.187	45.286	7.80E+07	1.17E+10	6.97E+05
P7.11	CRR2039295		61.871	100.836	20.114	120.002	1.203	144.389	9.84E+07	1.47E+10	6.83E+05
P7.12	CRR2039296		59.873	94.730	14.664	113.020	1.164	131.584	1.00E+08	1.50E+10	6.18E+05
P9.01	CRR2039297		55.108	67.423	17.014	88.726	1.182	104.875	8.45E+07	1.27E+10	7.05E+05
P9.02	CRR2039298		51.067	63.352	13.587	82.498	1.162	95.863	1.52E+08	2.28E+10	1.16E+06
P9.03	CRR2039299		60.547	115.175	17.230	131.256	1.181	154.996	7.84E+07	1.17E+10	6.34E+05
P9.04	CRR2039300		53.722	62.610	14.957	83.844	1.177	98.687	1.13E+08	1.69E+10	8.33E+05
P9.05	CRR2039301		60.362	75.561	17.276	98.242	1.188	116.723	9.78E+07	1.46E+10	7.42E+05
P9.06	CRR2039302		57.175	63.758	20.064	87.958	1.178	103.621	9.00E+07	1.35E+10	7.13E+05
P9.07	CRR2039303		47.294	46.421	13.892	67.710	1.176	79.626	8.89E+07	1.33E+10	7.36E+05

	P9.08	CRR2039304		56.010	48.127	18.886	76.223	1.194	91.033	9.04E+07	1.35E+10	7.04E+05		
	P9.09	CRR2039305		52.274	56.459	13.047	78.041	1.182	92.263	8.79E+07	1.31E+10	6.23E+05		
	P9.10	CRR2039306		49.715	55.430	16.783	76.326	1.182	90.218	8.01E+07	1.20E+10	6.11E+05		
	P9.11	CRR2039307		57.847	68.334	20.003	91.739	1.185	108.707	1.06E+08	1.59E+10	7.38E+05		
	P9.12	CRR2039308		52.676	70.214	15.138	89.073	1.173	104.452	9.55E+07	1.43E+10	7.49E+05		
wild animal feces (n=8)	AIA	SRR10492800	Wild animal feces collected from Qinghai Lake (3), AI= *Anser indicus*, BG= *Bos grunniens*, LI=*Larus ichthyaetus*, PC= *Phalacrocorax carbo*, SH=sheep, and bird feces collected from Yalujiang River (YR)	12.090	25.797	40.621	49.616	1.194	59.246	6.58E+07	9.52E+09	2.85E+05	30.971	19.163
	AIB	SRR10492799		2.854	14.470	31.014	34.342	1.183	40.617	6.47E+07	9.45E+09	2.95E+05		
	BG	SRR10492796		8.385	10.213	11.046	17.223	1.256	21.631	7.11E+07	1.01E+10	3.61E+05		
	LI	SRR10492798		17.635	24.909	40.595	50.788	1.091	55.416	6.22E+07	9.20E+09	3.27E+05		
	PC	SRR10492797		2.643	25.083	15.535	29.623	1.228	36.379	6.44E+07	9.26E+09	5.80E+04		
	SH	SRR10492795		1.996	0.744	2.657	3.405	1.159	3.948	6.43E+07	9.47E+09	4.99E+05		
	YRBE2	CRR2042949		0.454	6.491	3.719	7.495	1.058	7.931	5.54E+07	8.30E+09	7.62E+04		
	YRBE3	CRR2042950		0.639	1.879	15.397	15.525	1.456	22.600	5.51E+07	8.26E+09	3.43E+04		
wilderness environment (n=8)	QLFW	SRR10492803	Fresh water (FW), sand and soil (SS), salt water (SW) collected from Qinghai Lake (QL)(3) and Yancheng, Jiangsu Province, soil (S), water (W) collected from Yalujiang River (YR)	0.884	1.257	1.607	2.224	1.000	2.224	6.80E+07	1.02E+10	4.41E+05	2.603	1.775
	QLSS	SRR10492801		1.638	2.412	2.696	3.971	1.000	3.971	7.83E+07	1.17E+10	3.49E+05		
	QLSW	SRR10492802		0.018	3.710	0.199	3.715	1.000	3.715	7.30E+07	1.09E+10	4.79E+05		
	YRS	CRR2042957		0.012	0.551	0.242	0.602	1.030	0.620	4.74E+07	7.10E+09	3.00E+05		
	YRW	CRR2042954		0.317	5.798	0.135	5.809	1.047	6.083	5.28E+07	7.91E+09	3.55E+05		
	JYFW	CRR2042955		0.523	2.108	0.519	2.233	1.087	2.427	4.12E+07	6.17E+09	2.70E+05		
	JYSW	CRR2042956		0.133	0.698	0.085	0.716	1.071	0.766	4.11E+07	6.15E+09	2.57E+05		
	JYTS	CRR2042958		0.611	0.437	0.691	1.020	1.000	1.020	4.08E+07	6.11E+09	1.62E+05		

Table S5. Information of 240 short-read metagenomic samples collected from public databases. Detailed descriptions of all samples can be found at NCBI (https://www.ncbi.nlm.nih.gov/).

Environment	Project	References	Sample	Risk Vector			Risk Modulus	Cooccur Score	Risk Index	Number of Reads	Number of Bases	Number of Contigs	Mean of Risk Index	SD of Risk Index
				ARGs	MGE	VFs								
hospital	PRJEB31632	(4)	ERR3209754	59.370	201.787	8.328	210.505	1.314	276.677	2.89E+07	2.89E+09	3.60E+04	114.508	94.141
(n=46)			ERR3209755	73.939	258.882	3.172	269.252	1.385	372.977	2.64E+07	2.64E+09	9.80E+03		
			ERR3209803	71.356	177.869	20.233	192.714	1.423	274.190	3.17E+07	3.18E+09	3.11E+04		
			ERR3674529	0.889	0.791	0.210	1.209	1.103	1.333	4.42E+07	6.59E+09	6.28E+05		
			ERR3674536	11.583	13.576	18.514	25.714	1.271	32.682	4.89E+07	7.28E+09	2.02E+05		
			ERR3674560	43.783	299.328	5.681	302.566	1.312	396.996	5.38E+07	8.03E+09	3.04E+04		
			ERR3674565	2.070	3.654	3.988	5.792	1.213	7.022	5.79E+07	8.61E+09	7.07E+05		
			ERR3674566	2.708	23.427	19.375	30.521	1.339	40.866	4.44E+07	6.60E+09	8.87E+04		
			ERR3675723	7.859	31.281	1.379	32.282	1.133	36.574	5.42E+06	8.01E+08	1.63E+04		
			ERR3679653	6.690	37.449	7.775	38.828	1.067	41.430	1.05E+07	1.44E+09	5.22E+04		
			ERR3679665	2.591	98.777	8.449	99.171	1.190	118.002	3.01E+07	4.34E+09	7.46E+04		
			ERR3679666	0.258	3.155	0.013	3.166	1.092	3.456	1.65E+07	2.35E+09	1.69E+04		
			ERR3679670	4.993	35.088	17.619	39.579	1.078	42.655	2.71E+07	3.94E+09	1.64E+05		
			ERR3679675	2.205	42.002	2.755	42.150	1.152	48.550	2.91E+07	4.21E+09	8.31E+04		
	PRJNA1033037	(5)	SRR26638131	15.133	10.959	2.471	18.847	1.303	24.561	8.98E+07	1.32E+10	5.09E+05		
			SRR26638135	59.290	48.932	15.158	78.355	1.348	105.590	7.92E+07	1.16E+10	4.97E+05		
			SRR26638139	8.429	13.462	1.206	15.929	1.285	20.462	8.47E+07	1.25E+10	2.00E+05		
			SRR26638145	6.919	10.157	1.158	12.344	1.301	16.056	1.01E+08	1.48E+10	2.99E+05		
			SRR26638149	11.090	19.136	1.266	22.153	1.271	28.149	7.37E+07	1.08E+10	5.59E+05		
			SRR26638155	24.217	30.920	3.120	39.398	1.360	53.567	7.08E+07	1.05E+10	9.57E+04		
	PRJNA631351	(6)	SRR11745693	1.981	2.616	3.918	5.111	1.273	6.504	3.79E+07	5.10E+09	2.23E+05		
			SRR11745694	32.351	18.380	64.971	74.871	1.618	121.126	4.83E+07	6.62E+09	4.31E+05		
			SRR11745695	13.879	140.799	16.049	142.389	1.381	196.659	4.20E+07	5.82E+09	4.06E+04		
			SRR11745696	61.691	55.821	60.131	102.653	1.414	145.200	3.55E+07	5.05E+09	5.14E+04		
			SRR11745697	23.944	99.568	14.876	103.481	1.209	125.101	4.19E+07	5.50E+09	1.16E+05		

	PRJEB34690	(7)	ERR3569137	29.347	37.660	233.886	238.709	1.493	356.396	6.51E+07	6.51E+09	2.15E+04		
			ERR3569143	43.503	61.236	66.001	99.993	1.234	123.385	6.71E+07	6.71E+09	1.19E+05		
			ERR3569152	13.342	39.283	56.752	70.299	1.186	83.357	6.11E+07	6.11E+09	1.13E+05		
	PRJEB13831	(8)	ERR1713334	26.602	121.581	15.718	125.446	1.164	146.065	4.17E+07	6.28E+09	3.53E+05		
			ERR1713385	60.154	82.405	8.708	102.396	1.178	120.639	6.47E+07	9.73E+09	4.72E+05		
			ERR1725942	37.431	129.810	20.004	136.572	1.188	162.292	6.96E+07	1.05E+10	4.29E+05		
			ERR1725992	45.759	167.017	24.750	174.932	1.213	212.114	4.94E+07	7.45E+09	3.56E+05		
			ERR2592244	39.263	194.801	19.125	199.636	1.156	230.790	4.45E+07	6.72E+09	2.99E+05		
			ERR2592247	24.874	160.998	8.541	163.132	1.154	188.294	4.26E+07	6.43E+09	3.24E+05		
			ERR2592252	50.622	75.877	7.970	91.561	1.170	107.129	5.06E+07	7.63E+09	3.73E+05		
			ERR9855090	86.644	103.169	9.215	135.041	1.190	160.634	5.88E+07	8.53E+09	3.20E+05		
			ERR9855099	10.378	27.660	5.081	29.977	1.141	34.217	7.49E+07	1.09E+10	5.92E+05		
	PRJNA675192	(9)	SRR13003733	42.588	17.139	1.518	45.932	1.214	55.771	3.54E+07	5.30E+09	2.22E+05		
			SRR13003735	20.433	20.019	12.901	31.380	1.222	38.359	5.90E+07	8.84E+09	2.55E+05		
			SRR13003737	60.427	42.709	10.213	74.698	1.316	98.302	5.07E+07	7.61E+09	2.96E+05		
			SRR13003743	35.082	31.554	9.218	48.077	1.242	59.689	3.97E+07	5.94E+09	1.40E+05		
			SRR13003746	49.451	33.568	1.804	59.796	1.257	75.141	4.76E+07	7.13E+09	2.96E+05		
			SRR13003749	76.645	69.910	7.463	104.008	1.239	128.887	3.58E+07	5.36E+09	2.50E+05		
			SRR13003751	100.083	124.250	20.357	160.839	1.257	202.233	4.61E+07	6.91E+09	3.04E+05		
			SRR13003753	37.547	15.037	0.998	40.458	1.194	48.316	3.52E+07	5.28E+09	2.01E+05		
			SRR13003756	69.519	31.665	14.652	77.783	1.273	98.986	5.06E+07	7.58E+09	2.06E+05		
human feces (n=39)	PRJNA530971	(10)	SRR8849198	37.249	25.584	2.068	45.236	1.194	54.012	2.38E+07	5.95E+09	8.67E+04	99.942	53.686
			SRR8849201	59.465	18.298	11.106	63.200	1.365	86.249	2.17E+07	5.42E+09	7.90E+04		
			SRR8849204	60.763	31.044	4.158	68.360	1.232	84.222	3.07E+07	7.68E+09	6.71E+04		
			SRR8849207	62.656	19.438	14.262	67.134	1.388	93.203	2.22E+07	5.55E+09	7.92E+04		
			SRR8849210	49.646	17.846	21.412	56.935	1.455	82.867	6.66E+07	9.98E+09	7.48E+04		
			SRR8849213	53.332	10.389	9.162	55.102	1.376	75.840	7.86E+07	1.18E+10	8.77E+04		
			SRR8849216	34.640	24.631	0.114	42.504	1.118	47.511	6.52E+07	9.78E+09	8.54E+04		
			SRR8849219	43.086	16.003	4.500	46.181	1.257	58.063	2.28E+07	5.69E+09	1.13E+05		

		SRR8849222	57.235	18.701	22.388	64.240	1.353	86.895	6.54E+07	9.79E+09	1.07E+05
		SRR8849225	99.387	35.878	16.187	106.897	1.525	162.999	6.47E+07	9.68E+09	3.63E+04
		SRR8849228	51.071	13.256	6.779	53.197	1.432	76.194	7.11E+07	1.07E+10	1.11E+05
		SRR8849231	48.682	40.557	4.521	63.524	1.360	86.385	7.08E+07	1.06E+10	6.36E+04
PRJNA820119	(11)	SRR18491229	49.897	27.032	0.857	56.755	1.183	67.117	4.34E+07	6.48E+09	1.18E+05
		SRR18491224	49.308	23.352	3.258	54.655	1.265	69.124	6.07E+07	9.02E+09	1.67E+05
		SRR18491219	61.768	24.525	16.961	68.589	1.401	96.118	3.91E+07	5.85E+09	3.94E+04
		SRR18491214	53.706	20.987	14.266	59.400	1.441	85.592	7.06E+07	1.06E+10	6.62E+04
		SRR18491209	45.655	21.856	6.229	50.999	1.263	64.406	5.90E+07	8.83E+09	1.34E+05
		SRR18491234	43.657	18.015	0.614	47.232	1.148	54.202	5.72E+07	8.54E+09	1.23E+05
		SRR18491235	52.090	17.449	11.266	56.078	1.469	82.360	4.07E+07	6.07E+09	4.49E+04
		SRR18491241	59.988	25.937	14.260	66.893	1.368	91.542	7.59E+07	1.14E+10	1.84E+05
		SRR18491246	51.561	13.668	1.050	53.352	1.149	61.312	5.57E+07	8.34E+09	1.36E+05
		SRR18491251	154.137	47.406	12.202	161.724	1.256	203.177	4.86E+07	7.28E+09	1.02E+05
		SRR18491256	37.894	10.610	0.230	39.352	1.120	44.070	5.60E+07	8.32E+09	1.22E+05
		SRR18491261	43.798	10.934	5.635	45.492	1.278	58.155	5.30E+07	7.94E+09	1.47E+05
PRJNA906498	(12)	SRR22519754	39.623	12.750	3.285	41.754	1.208	50.450	3.45E+07	5.18E+09	1.30E+05
		SRR22519762	82.183	54.889	41.400	107.149	1.431	153.282	3.38E+07	5.07E+09	3.86E+04
		SRR22519770	30.723	14.478	9.468	35.258	1.280	45.119	3.43E+07	5.15E+09	7.64E+04
		SRR22519778	42.629	32.737	9.939	54.660	1.327	72.520	4.31E+07	6.47E+09	5.20E+04
		SRR22519786	46.421	28.401	22.618	58.933	1.602	94.397	3.52E+07	5.28E+09	3.23E+04
		SRR22519794	167.240	73.646	160.135	242.974	1.416	343.968	3.32E+07	4.98E+09	1.07E+05
PRJNA1039317	(13)	SRR26803178	67.679	27.258	1.236	72.973	1.177	85.907	4.50E+07	6.76E+09	1.29E+05
		SRR26803197	50.004	25.978	4.151	56.502	1.284	72.540	4.51E+07	6.76E+09	1.01E+05
		SRR26803208	43.726	29.287	0.138	52.628	1.118	58.813	4.40E+07	6.61E+09	6.93E+04
		SRR26803219	62.670	20.839	0.965	66.051	1.197	79.039	4.48E+07	6.73E+09	4.94E+04
		SRR26803261	79.676	46.607	6.658	92.546	1.359	125.757	4.48E+07	6.72E+09	9.47E+04
		SRR26803272	46.566	25.987	14.020	55.139	1.287	70.954	4.35E+07	6.52E+09	1.04E+05
		SRR26803283	112.525	51.438	9.655	124.101	1.327	164.713	4.00E+07	5.99E+09	6.42E+04

			SRR26803332	67.979	20.118	6.757	71.214	1.479	105.319	4.70E+07	7.05E+09	7.98E+04		
			SRR26803334	39.780	23.858	0.114	46.386	1.128	52.330	4.49E+07	6.73E+09	7.30E+04		
municipal sewage (n=35)	PRJEB64323	(14)	ERR13912360	8.493	15.621	53.511	56.388	1.166	65.752	6.21E+07	9.28E+09	2.62E+05	51.819	28.845
			ERR13912361	10.513	20.472	63.655	67.687	1.206	81.645	8.05E+07	1.20E+10	1.37E+05		
			ERR13912362	8.647	21.166	43.491	49.135	1.188	58.362	8.81E+07	1.32E+10	3.77E+05		
			ERR13912363	8.666	22.752	36.388	43.781	1.266	55.436	1.66E+07	2.48E+09	8.13E+04		
			ERR13912364	3.170	3.828	6.354	8.067	1.128	9.097	1.29E+07	1.94E+09	7.34E+04		
			ERR13912365	9.632	84.093	13.525	85.716	1.156	99.105	2.38E+07	3.57E+09	1.63E+05		
			ERR13912366	3.525	24.484	0.777	24.749	1.101	27.254	4.18E+07	6.23E+09	2.70E+05		
	PRJNA505617	(15)	SRR8208343	22.213	67.747	23.687	75.127	1.156	86.844	1.18E+08	1.77E+10	9.26E+05		
			SRR8208344	33.374	64.356	12.394	73.547	1.179	86.701	1.10E+08	1.65E+10	7.87E+05		
			SRR8208347	23.148	67.228	8.966	71.664	1.141	81.748	3.77E+07	5.65E+09	2.00E+05		
	PRJNA1196808	(16)	SRR31677053	10.273	18.466	93.347	95.709	1.147	109.734	8.68E+07	1.25E+10	1.56E+05		
			SRR31677055	6.304	17.558	67.478	70.010	1.145	80.177	6.75E+07	9.77E+09	1.34E+05		
			SRR31677056	0.893	7.870	4.994	9.363	1.075	10.062	6.71E+07	9.79E+09	1.56E+05		
			SRR31677058	4.484	18.981	50.573	54.203	1.203	65.213	6.98E+07	1.00E+10	7.89E+04		
	PRJNA661613	N.A.	SRR23998354	12.744	36.479	12.192	40.519	1.149	46.543	8.02E+07	5.90E+09	3.49E+04		
			SRR23998356	11.062	29.134	4.903	31.547	1.179	37.184	9.33E+07	6.83E+09	6.38E+04		
			SRR23998357	10.551	22.704	8.140	26.326	1.191	31.354	9.45E+07	6.92E+09	4.96E+04		
	PRJEB38014	(17)	ERR4450742	8.187	9.113	9.312	15.388	1.325	20.389	6.11E+07	6.11E+09	1.03E+05		
			ERR4450744	44.722	62.630	9.906	77.594	1.214	94.206	6.16E+07	6.16E+09	1.90E+05		
			ERR4450752	55.732	32.268	4.731	64.573	1.252	80.825	6.18E+07	6.18E+09	1.98E+05		
			ERR4450754	55.846	33.292	7.865	65.490	1.264	82.799	6.19E+07	6.19E+09	1.96E+05		
			ERR4450756	2.594	1.945	0.540	3.287	1.197	3.935	6.01E+07	6.01E+09	1.43E+05		
			ERR4450758	25.361	28.793	4.116	38.590	1.186	45.765	6.09E+07	6.08E+09	1.73E+05		
			ERR4450760	42.162	54.089	3.822	68.687	1.144	78.555	6.39E+07	6.39E+09	1.53E+05		
			ERR4450763	17.007	16.757	1.667	23.933	1.139	27.253	5.63E+07	5.62E+09	1.63E+05		
			ERR4450765	15.216	12.491	1.176	19.721	1.201	23.686	6.05E+07	6.05E+09	1.92E+05		
	PRJNA1176457	N.A.	SRR31091721	0.426	14.168	1.802	14.289	1.091	15.588	4.40E+07	6.64E+09	2.11E+05		

			SRR31091722	4.362	25.611	3.525	26.218	1.122	29.410	5.62E+07	8.47E+09	3.00E+05		
			SRR31091725	1.513	45.359	2.157	45.436	1.110	50.442	4.63E+07	6.97E+09	2.29E+05		
	PRJEB81865	N.A.	ERR13925543	27.095	24.949	2.083	36.891	1.132	41.750	6.14E+07	9.10E+09	3.54E+05		
			ERR13925542	28.477	37.745	21.228	51.830	1.263	65.447	6.70E+07	1.00E+10	3.38E+05		
			ERR13925545	30.834	22.700	4.021	38.500	1.140	43.873	7.53E+07	1.12E+10	5.48E+05		
	PRJNA505617	(15)	SRR8206192	6.428	16.355	13.433	22.119	1.113	24.629	8.77E+07	1.31E+10	5.04E+05		
			SRR8208346	5.547	16.243	13.025	21.547	1.095	23.594	9.00E+07	1.35E+10	6.15E+05		
			SRR8208349	6.967	21.702	10.735	25.194	1.163	29.304	8.87E+07	1.33E+10	5.41E+05		
treated sewage (n=35)	PRJNA847265	N.A.	SRR19591708	3.047	23.956	5.133	24.688	1.092	26.954	5.62E+07	8.49E+09	4.11E+05	13.234	11.233
			SRR19591712	1.194	4.837	2.673	5.654	1.076	6.087	5.33E+07	8.05E+09	4.78E+05		
			SRR19591716	3.486	9.453	4.606	11.078	1.105	12.246	4.63E+07	6.99E+09	3.65E+05		
	PRJNA623645	(18)	SRR12348546	2.709	8.172	1.207	8.693	1.072	9.319	5.95E+07	8.92E+09	3.37E+05		
			SRR12348547	4.978	7.078	1.758	8.830	1.108	9.783	6.83E+07	1.02E+10	5.99E+05		
			SRR12348548	5.569	5.126	2.987	8.137	1.095	8.909	5.63E+07	8.44E+09	4.47E+05		
			SRR12348549	2.674	3.257	1.248	4.394	1.094	4.806	6.96E+07	1.04E+10	6.47E+05		
			SRR12348550	3.618	3.461	1.458	5.215	1.099	5.733	6.65E+07	9.95E+09	5.78E+05		
	PRJDB14461	N.A.	DRR438448	3.785	8.351	8.533	12.525	1.105	13.835	2.63E+08	3.94E+10	3.78E+05		
			DRR438457	5.151	17.446	15.297	23.768	1.168	27.751	2.07E+08	3.11E+10	3.12E+05		
			DRR438466	3.602	20.202	7.791	21.949	1.145	25.124	2.03E+08	3.04E+10	2.41E+05		
			DRR438475	4.735	25.812	12.257	28.964	1.101	31.877	2.25E+08	3.37E+10	6.91E+05		
			DRR438484	5.818	39.165	5.770	40.013	1.123	44.926	2.04E+08	3.07E+10	7.12E+05		
			DRR438493	0.217	14.538	0.826	14.563	1.064	15.499	1.83E+08	2.75E+10	5.63E+05		
			DRR438511	1.620	27.561	6.195	28.295	1.141	32.279	2.49E+08	3.74E+10	2.37E+05		
			DRR438524	0.751	22.229	5.547	22.923	1.124	25.764	2.63E+08	3.94E+10	2.82E+05		
			DRR438536	6.762	20.966	10.029	24.205	1.092	26.443	2.66E+08	3.98E+10	1.60E+06		
	PRJNA1196808	(16)	SRR31677059	1.017	1.125	0.054	1.517	1.074	1.629	6.78E+07	9.58E+09	5.99E+04		
			SRR31677061	0.377	0.172	0.000	0.414	1.667	0.691	2.52E+07	3.56E+09	1.34E+04		
			SRR31677062	0.209	0.370	0.028	0.426	1.188	0.506	2.59E+07	3.63E+09	1.41E+04		
			SRR31677063	0.225	1.183	0.141	1.213	1.041	1.263	8.30E+07	1.19E+10	5.76E+04		

	PRJEB26809	(19)	ERR2586215	7.517	12.260	2.813	14.654	1.178	17.258	5.38E+07	6.77E+09	2.00E+05		
			ERR2586216	6.771	11.796	2.147	13.770	1.195	16.454	5.74E+07	7.23E+09	1.98E+05		
			ERR2586217	6.663	11.802	2.822	13.843	1.168	16.171	6.53E+07	8.23E+09	2.34E+05		
			ERR2586218	3.806	10.177	2.100	11.066	1.089	12.054	5.19E+07	6.53E+09	4.02E+05		
			ERR2586219	3.665	9.821	1.881	10.650	1.081	11.510	5.99E+07	7.54E+09	4.63E+05		
			ERR2586220	2.752	10.050	1.694	10.557	1.081	11.413	4.92E+07	6.19E+09	3.82E+05		
	PRJNA706754	(20)	SRR14120362	0.697	3.743	0.296	3.819	1.099	4.198	5.36E+07	7.76E+09	2.95E+05		
			SRR14120364	0.089	0.483	0.077	0.497	1.055	0.524	6.44E+07	9.44E+09	3.93E+05		
			SRR14120366	1.837	12.611	1.039	12.787	1.131	14.465	5.83E+07	8.34E+09	2.84E+05		
			SRR14120368	0.500	0.877	0.973	1.402	1.106	1.550	6.16E+07	8.98E+09	3.26E+05		
			SRR14120372	0.000	0.534	0.456	0.702	1.033	0.725	6.05E+07	8.78E+09	4.48E+05		
			SRR14120374	0.368	0.259	0.807	0.924	1.021	0.943	7.57E+07	1.10E+10	5.49E+05		
			SRR14120376	3.468	20.758	4.217	21.464	1.111	23.839	6.03E+07	8.73E+09	3.28E+05		
			SRR14120378	0.090	0.497	0.420	0.657	1.035	0.680	5.88E+07	8.42E+09	3.01E+05		
activated sludge (n=29)	PRJNA949407	(21)	SRR23986424	8.803	26.138	6.963	28.445	1.136	32.318	1.06E+08	1.60E+10	1.50E+05	11.443	7.219
			SRR23986425	8.384	25.800	5.993	27.782	1.122	31.159	1.04E+08	1.57E+10	1.53E+05		
	PRJNA295114	N.A.	SRR2308827	2.576	12.083	1.805	12.485	1.083	13.526	5.06E+07	5.10E+09	1.90E+05		
			SRR2308974	1.594	6.281	1.758	6.715	1.059	7.108	5.18E+07	5.23E+09	1.49E+05		
	PRJEB48021	(22)	ERR7015307	4.136	3.961	0.071	5.727	1.061	6.075	4.65E+07	1.31E+10	4.33E+05		
			ERR7015405	2.897	4.834	0.028	5.636	1.065	6.003	2.05E+07	4.89E+09	2.21E+05		
			ERR7015688	2.640	4.571	0.284	5.286	1.064	5.622	2.80E+07	6.66E+09	2.54E+05		
	PRJNA432264	(23)	SRR6747722	0.654	5.943	3.529	6.943	1.078	7.486	4.95E+07	7.41E+09	5.26E+05		
			SRR6747732	1.051	8.426	4.731	9.720	1.047	10.178	3.14E+07	4.71E+09	3.59E+05		
			SRR6747742	1.091	9.607	6.261	11.519	1.061	12.221	4.29E+07	6.42E+09	4.39E+05		
			SRR6747752	2.081	13.027	4.913	14.078	1.044	14.695	2.94E+07	4.41E+09	3.19E+05		
			SRR6747762	6.185	12.450	12.099	18.430	1.064	19.609	3.25E+07	4.87E+09	3.49E+05		
			SRR6747772	2.106	10.756	5.612	12.313	1.062	13.078	3.85E+07	5.78E+09	4.09E+05		
			SRR6747782	0.029	0.368	0.006	0.369	1.063	0.393	9.90E+07	1.48E+10	2.80E+05		
			SRR6747792	1.303	6.497	1.311	6.755	1.081	7.304	2.74E+07	4.11E+09	2.95E+05		

			SRR6747802	5.589	13.732	16.504	22.185	1.059	23.493	4.26E+07	6.38E+09	3.66E+05		
			SRR6747812	1.464	5.327	2.416	6.030	1.052	6.341	3.71E+07	5.57E+09	4.19E+05		
	PRJNA629478	(24)	SRR11674000	0.168	4.021	1.869	4.437	1.062	4.711	3.03E+07	4.51E+09	1.10E+05		
			SRR11674002	2.423	10.509	5.641	12.171	1.053	12.820	1.93E+08	2.85E+10	1.31E+06		
			SRR11674042	0.902	4.344	1.235	4.606	1.080	4.973	2.76E+07	4.07E+09	1.32E+05		
			SRR11674044	1.751	10.304	4.541	11.395	1.065	12.134	1.92E+08	2.81E+10	1.22E+06		
			SRR11674046	0.857	6.790	1.928	7.110	1.054	7.497	4.28E+07	6.32E+09	2.22E+05		
			SRR11674048	2.708	13.351	3.645	14.102	1.075	15.163	1.95E+08	2.86E+10	1.20E+06		
			SRR11674050	1.396	6.722	1.402	7.007	1.066	7.471	3.29E+07	4.79E+09	1.46E+05		
			SRR11674052	0.445	6.948	1.280	7.079	1.054	7.462	2.92E+07	4.32E+09	1.26E+05		
			SRR11674054	1.582	9.406	3.247	10.076	1.069	10.769	1.75E+08	2.46E+10	8.84E+05		
	PRJNA505617	(15)	SRR8205411	3.119	9.360	4.868	11.002	1.090	11.989	1.05E+08	1.58E+10	7.98E+05		
			SRR8208345	0.881	7.546	1.032	7.667	1.056	8.094	1.34E+07	2.01E+09	1.08E+05		
			SRR8208348	2.529	7.805	7.549	11.149	1.089	12.143	9.87E+07	1.48E+10	8.35E+05		
common soil and water (n=41)	PRJNA468911	N.A.	SRR7686822	1.300	1.512	0.368	2.028	1.179	2.392	2.82E+07	4.22E+09	2.32E+05	7.216	5.663
			SRR7686823	0.104	5.865	0.403	5.880	1.171	6.888	2.59E+07	3.88E+09	2.28E+05		
			SRR7686824	0.014	1.736	0.149	1.743	1.176	2.049	2.88E+07	4.31E+09	2.34E+05		
			SRR7686826	0.918	2.010	0.220	2.221	1.180	2.621	2.92E+07	4.37E+09	2.57E+05		
			SRR7686830	0.881	2.735	0.523	2.921	1.127	3.291	2.87E+07	4.29E+09	2.60E+05		
			SRR7686834	0.465	2.262	0.745	2.427	1.068	2.592	2.54E+07	3.79E+09	2.32E+05		
			SRR7686838	0.736	7.077	0.231	7.119	1.171	8.335	2.77E+07	4.16E+09	2.27E+05		
			SRR7686842	0.513	4.881	0.495	4.933	1.146	5.655	3.11E+07	4.66E+09	2.57E+05		
			SRR7686846	0.924	4.628	0.451	4.741	1.142	5.414	2.99E+07	4.47E+09	2.35E+05		
			SRR7686850	0.308	6.447	0.296	6.461	1.260	8.140	2.73E+07	4.09E+09	1.84E+05		
	PRJNA891434	N.A.	SRR21941333	5.905	8.832	10.542	14.967	1.079	16.147	1.11E+08	1.57E+10	5.14E+05		
			SRR21941336	4.091	8.095	17.383	19.607	1.094	21.441	8.34E+07	1.20E+10	5.32E+05		
			SRR21941339	5.486	18.169	15.919	24.771	1.098	27.196	1.03E+08	1.48E+10	5.27E+05		
			SRR21941340	4.591	9.700	10.340	14.903	1.096	16.326	1.39E+08	1.92E+10	6.59E+05		
			SRR21941342	2.007	0.621	5.336	5.735	1.022	5.861	1.10E+08	1.52E+10	3.76E+05		

			SRR21941344	2.991	5.332	4.575	7.635	1.083	8.272	1.07E+08	1.50E+10	3.05E+05		
	PRJNA648365	(25)	SRR12316794	2.300	0.429	1.590	2.829	1.024	2.897	7.46E+07	1.10E+10	3.61E+05		
	PRJEB41174	(26)	ERR4837082	0.018	4.158	0.599	4.201	1.033	4.340	6.23E+07	7.19E+09	2.84E+05		
			ERR4837087	1.116	1.004	1.425	2.070	1.011	2.093	5.08E+07	5.73E+09	2.04E+05		
			ERR4837092	0.535	0.590	0.679	1.046	1.027	1.075	7.75E+07	9.03E+09	1.67E+05		
			ERR4837097	0.574	2.219	1.025	2.511	1.020	2.561	7.11E+07	8.60E+09	2.35E+05		
			ERR4837102	0.755	4.096	5.908	7.229	1.040	7.518	7.65E+07	8.86E+09	2.15E+05		
			ERR4837107	0.000	3.177	0.080	3.178	1.037	3.296	1.75E+07	1.74E+09	4.34E+04		
			ERR4837112	0.909	0.309	1.274	1.595	1.000	1.595	2.14E+07	2.13E+09	2.84E+04		
			ERR4837117	0.532	0.477	0.762	1.045	1.000	1.045	5.65E+07	5.61E+09	4.56E+04		
			ERR4837127	0.000	0.174	0.005	0.174	1.000	0.174	2.88E+07	2.86E+09	8.68E+03		
			ERR4837137	0.874	2.280	1.793	3.030	1.034	3.134	7.98E+07	1.12E+10	3.86E+05		
			ERR4837146	1.423	2.168	1.074	2.807	1.034	2.901	6.80E+07	9.56E+09	3.26E+05		
	PRJNA796691	N.A.	SRR17642938	5.431	1.137	5.540	7.841	1.021	8.003	9.23E+07	1.38E+10	5.03E+05		
			SRR17642941	6.774	1.780	7.575	10.317	1.012	10.445	9.19E+07	1.38E+10	5.97E+05		
			SRR17642944	4.666	1.039	5.310	7.145	1.023	7.305	8.06E+07	1.21E+10	4.08E+05		
			SRR17642947	3.080	1.214	4.451	5.548	1.016	5.635	9.60E+07	1.44E+10	4.97E+05		
			SRR17642950	5.640	1.015	7.356	9.325	1.005	9.371	9.25E+07	1.39E+10	5.57E+05		
			SRR17642953	5.882	1.762	7.700	9.849	1.007	9.915	9.23E+07	1.38E+10	6.58E+05		
	PRJNA818834	N.A.	SRR18494403	4.635	1.532	8.007	9.378	1.046	9.811	1.15E+08	1.74E+10	6.43E+05		
			SRR18494407	2.910	2.518	9.397	10.155	1.078	10.948	1.09E+08	1.65E+10	7.42E+05		
			SRR18494411	2.933	2.045	10.231	10.838	1.047	11.345	1.03E+08	1.55E+10	5.74E+05		
			SRR18494415	5.619	1.046	11.046	12.437	1.042	12.960	1.12E+08	1.69E+10	5.17E+05		
	PRJNA933579	N.A.	SRR23403438	1.250	5.111	1.727	5.538	1.088	6.023	4.98E+07	7.47E+09	3.49E+05		
			SRR23403440	0.616	2.581	3.005	4.009	1.212	4.860	4.75E+07	7.11E+09	2.47E+05		
			SRR23403442	1.055	4.036	10.470	11.271	1.243	14.004	4.99E+07	7.48E+09	2.36E+05		
bird feces	PRJNA890321	(27)	SRR21898906	68.927	56.787	17.738	91.052	1.272	115.797	8.28E+07	1.24E+10	7.98E+04	60.446	41.763
(n=3)			SRR21898909	6.720	4.672	10.859	13.598	1.098	14.928	8.00E+07	1.20E+10	5.62E+05		
			SRR21898922	26.789	21.311	23.998	41.806	1.211	50.612	8.61E+07	1.29E+10	2.85E+05		

livestock feces and wastewater (n=12)	PRJNA634981	N.A.	SRR11845619	32.906	14.615	9.228	37.169	1.176	43.720	9.08E+07	1.36E+10	5.11E+05	121.654	64.067
			SRR11845622	149.499	56.882	8.083	160.159	1.198	191.867	1.01E+08	1.52E+10	7.24E+05		
			SRR11845624	49.936	6.599	0.068	50.370	1.252	63.054	7.86E+07	1.18E+10	5.37E+05		
			SRR11845625	50.375	7.235	0.884	50.900	1.230	62.589	1.05E+08	1.58E+10	6.78E+05		
			SRR11845629	44.429	7.620	0.155	45.078	1.223	55.144	9.15E+07	1.37E+10	5.02E+05		
			SRR11845631	129.696	39.836	1.778	135.688	1.196	162.245	8.93E+07	1.34E+10	4.89E+05		
			SRR11845632	128.513	39.232	1.425	134.375	1.206	162.074	6.31E+07	9.47E+09	3.61E+05		
			SRR11845635	52.819	9.929	0.349	53.745	1.203	64.641	8.85E+07	1.33E+10	6.32E+05		
			SRR11845636	52.118	10.797	0.520	53.227	1.204	64.094	9.20E+07	1.38E+10	6.43E+05		
	PRJNA1125297	(28)	SRR29457783	161.086	50.972	13.626	169.506	1.176	199.327	1.36E+08	1.98E+10	3.95E+05		
			SRR29457802	144.336	72.923	4.653	161.778	1.164	188.238	1.95E+08	2.92E+10	5.91E+05		
			SRR29457804	160.303	49.870	3.239	167.912	1.208	202.854	1.27E+08	1.85E+10	6.26E+05		

Table S6. Information of 10 long-read metagenomic samples collected from public databases. Detailed descriptions of all samples can be found at NCBI (https://www.ncbi.nlm.nih.gov/).

Environment	Project	References	Platform	Sample	Risk Vector			Risk Modulus	Cooccur Score	Risk Index	Number of Reads	Number of Bases	Number of Contigs
					ARGs	MGE	VFs						
anaerobic digester at Fredericia WWTP	PRJEB48021	(22)	MinION	ERR7014844	4.5911	3.0251	0.0168	5.4982	1.2662	6.9618	2.40E+06	8.91E+09	12300
			PACBIO_SMRT (Sequel)	ERR7015089	3.9315	4.3573	0.0193	5.8688	1.4198	8.3329	9.93E+05	1.53E+10	7113
			GridION	ERR7256374	0.0000	0.0000	0.0007	0.0007	1.3070	0.0010	2.51E+06	1.05E+10	17983
activated sludge	PRJNA629478	(24)	PromethION	SRR11673988	0.3380	8.3721	0.1787	8.3808	1.3793	11.5598	2.16E+06	1.39E+10	21634
cow feces	PRJNA1162230	N.A.	PromethION	SRR30693771	10.2611	8.3937	0.0010	13.2569	1.8710	24.8032	2.66E+05	1.58E+09	3188
			PromethION	SRR30693871	8.5204	6.9786	0.0036	11.0135	1.5652	17.2386	2.26E+05	1.35E+09	2863
human feces	PRJNA1139951	N.A.	PromethION	SRR29980939	0.0000	9.4809	0.1329	9.4818	2.1317	20.2122	1.43E+06	1.99E+10	1807
			PromethION	SRR29980957	8.6934	4.1834	0.0175	9.6476	1.8425	17.7754	8.27E+05	4.94E+09	1932
			PACBIO_SMRT (Revio)	SRR29980922	36.4659	8.7544	2.4081	37.5793	2.0559	77.2579	4.15E+06	3.00E+10	6994
Seawater	PRJNA1220790	N.A.	PACBIO_SMRT (Sequel IIe)	SRR32280818	0.1219	0.1371	0.0311	0.1860	1.3333	0.2480	8.36E+05	9.48E+09	8436

Table S7. Information of CD-HIT cluster parameters test. High risk sample SRR22519794 and low risk sample SRR7686823 were selected to validate on different CD-HIT cluster identity and coverage from 1 to 0.7. According to the results below, a lower cluster identity and coverage will significantly reduce the runtime, but may increase the False Negative Rate of quantification (see Risk Modulus), increase the False Negative Rate of identification in higher risk samples and increase the False Positive Rate of identification in lower risk samples (see Cooccur Score). Thus, we chose 0.85 as the CD-HIT cluster identity and coverage, to balance the accuracy and time consumption.

Sample Name	CD-HIT Cluster Identity and Coverage	Risk Vector			Risk Modulus	Cooccur Score	Risk Index	Runtime (s)	Number of Reads	Number of Bases	Number of Contigs
		ARGs	MGEs	VFs							
SRR22519794 (human feces)	1	167.2565	74.6096	160.9258	243.8000	1.4208	346.3935	742.50	3.32E+07	4.98E+09	1.07E+05
	0.9	167.2543	74.3055	160.8128	243.6310	1.4174	345.3322	699.93			
	0.85	167.2399	73.6461	160.1353	242.9738	1.4157	343.9676	643.33			
	0.8	167.0866	71.7598	155.9814	239.5779	1.4015	335.7687	625.83			
	0.7	140.1331	69.8531	140.0511	210.0739	1.3811	290.1325	607.10			
SRR7686823 (common soil and water)	1	0.1040	5.8957	0.4389	5.9129	1.1552	6.8304	715.20	2.59E+07	3.88E+09	2.28E+05
	0.9	0.1040	5.8669	0.4034	5.8817	1.1667	6.8620	679.74			
	0.85	0.1040	5.8649	0.4031	5.8797	1.1714	6.8876	632.37			
	0.8	0.1040	5.8544	0.2157	5.8592	1.1856	6.9465	618.63			
	0.7	0.1040	5.7575	0.0962	5.7593	1.2222	7.0391	604.35			

Table S8. Information of MetaRanker's database construction process. Most redundant sequences were removed during the CD-HIT clustering step. Several overlapping segments (VFs vs. ARGs, VFs vs. MGEs, ARGs vs. MGEs) were removed in the subsequent refining step.

Database	Number of Sequences			Number of Overlapping Segments	
	Raw	After CD-HIT (Redundancy proportion)	After Overlap-Removing (Removed Proportion)	VFs Segments (in How Many Sequences)	ARGs Segments (in How Many Sequences)
VFDB	32674	14318 (56.18%)	14318 (0%)	-	-
CARD	6048	1218 (79.86%)	1207 (0.90%)	11 (11)	-
MGE	6808	5725 (15.91%)	5692 (0.58%)	38 (24)	1030 (202)

Table S9. Information of 20 mock samples used for benchmarking.

Group	Sample Name	Pollution Level	Risk Vector			Risk Modulus	Cooccur Score	Risk Index	Number of Reads	Number of Bases	Number of Contigs
			ARGs	MGEs	VFs						
SRR22519794+QLFW	QLFW	0	0.884	1.257	1.607	2.224	1.000	2.224	6.80E+07	1.02E+10	4.41E+05
	SRR22519794+QLFW_1_25	0.140	22.338	10.852	22.339	33.403	1.382	46.175	5.93E+07	8.92E+09	3.55E+05
	SRR22519794+QLFW_2_25		23.271	10.601	20.799	32.962	1.368	45.093	5.93E+07	8.92E+09	3.54E+05
	SRR22519794+QLFW_3_25		23.578	10.421	21.707	33.700	1.388	46.777	5.93E+07	8.92E+09	3.54E+05
	SRR22519794+QLFW_1_50	0.328	54.805	24.226	50.481	78.351	1.401	109.753	5.06E+07	7.61E+09	2.78E+05
	SRR22519794+QLFW_2_50		55.093	24.495	50.436	78.607	1.389	109.194	5.06E+07	7.61E+09	2.78E+05
	SRR22519794+QLFW_3_50		54.326	24.448	50.585	78.153	1.400	109.414	5.06E+07	7.61E+09	2.78E+05
	SRR22519794+QLFW_1_75	0.595	99.657	42.748	90.769	141.414	1.429	202.074	4.19E+07	6.30E+09	2.01E+05
	SRR22519794+QLFW_2_75		98.842	43.849	88.764	139.898	1.433	200.425	4.19E+07	6.30E+09	2.01E+05
	SRR22519794+QLFW_3_75		99.106	43.575	91.756	141.915	1.410	200.080	4.19E+07	6.30E+09	2.01E+05
	SRR22519794	1	167.240	73.646	160.135	242.974	1.416	343.968	3.32E+07	4.98E+09	1.07E+05
HRG+LRG	LRG_1_00	0	3.235	7.549	2.686	8.641	1.044	9.020	5.99E+07	8.99E+09	2.31E+04
	LRG_2_00		3.580	7.645	2.176	8.717	1.034	9.010	5.99E+07	8.99E+09	2.30E+04
	LRG_3_00		2.262	7.360	1.894	7.929	1.026	8.132	6.00E+07	8.99E+09	2.31E+04
	HRG+LRG_1_25	0.249	166.739	85.066	134.325	230.394	1.550	357.191	5.98E+07	8.97E+09	5.04E+04
	HRG+LRG_2_25		165.119	84.644	134.699	229.287	1.547	354.762	5.98E+07	8.97E+09	5.03E+04
	HRG+LRG_3_25		168.137	85.527	134.263	231.541	1.560	361.280	5.98E+07	8.98E+09	5.03E+04
	HRG+LRG_1_50	0.498	326.972	162.686	265.345	451.426	1.553	700.914	5.97E+07	8.96E+09	5.03E+04
	HRG+LRG_2_50		328.955	164.137	266.577	454.110	1.545	701.607	5.97E+07	8.96E+09	5.03E+04
	HRG+LRG__50		329.797	163.608	266.527	454.500	1.555	706.816	5.97E+07	8.96E+09	5.02E+04
	HRG+LRG_1_75	0.749	491.276	242.297	396.907	676.458	1.547	1046.245	5.96E+07	8.94E+09	5.08E+04
	HRG+LRG_2_75		491.396	240.625	397.345	676.206	1.549	1047.711	5.96E+07	8.94E+09	5.08E+04
	HRG+LRG_3_75		489.117	239.994	399.962	675.871	1.550	1047.560	5.96E+07	8.94E+09	5.08E+04
	HRG_1_100	1	656.954	321.771	529.455	903.022	1.569	1417.003	5.95E+07	8.93E+09	2.69E+04
	HRG_2_100		660.278	321.581	528.676	904.920	1.566	1417.520	5.95E+07	8.92E+09	2.71E+04
	HRG_3_100		657.818	320.726	535.787	907.005	1.569	1423.265	5.95E+07	8.93E+09	2.70E+04
SRR22519794+LRG	LRG_1_00	0	3.235	7.549	2.686	8.641	1.044	9.020	5.99E+07	8.99E+09	2.31E+04
	LRG_2_00		3.580	7.645	2.176	8.717	1.034	9.010	5.99E+07	8.99E+09	2.30E+04
	LRG_3_00		2.262	7.360	1.894	7.929	1.026	8.132	6.00E+07	8.99E+09	2.31E+04
	SRR22519794+LRG_1_25	0.156	28.949	17.317	26.239	42.737	1.452	62.073	5.33E+07	7.99E+09	8.34E+04
	SRR22519794+LRG_2_25		29.161	17.536	25.641	42.607	1.435	61.149	5.33E+07	7.99E+09	8.34E+04
	SRR22519794+LRG_3_25		28.353	17.592	25.509	42.001	1.411	59.263	5.33E+07	7.99E+09	8.35E+04
	SRR22519794+LRG_1_50	0.357	61.445	30.271	56.040	88.500	1.424	126.010	4.66E+07	6.99E+09	1.05E+05
	SRR22519794+LRG_2_50		61.579	30.739	56.127	88.810	1.435	127.433	4.66E+07	6.99E+09	1.06E+05
	SRR22519794+LRG_3_50		60.941	30.426	56.228	88.324	1.396	123.292	4.66E+07	6.99E+09	1.05E+05

	SRR22519794+LRG_1_75	0.624	106.976	48.151	95.184	151.071	1.421	214.679	3.99E+07	5.99E+09	1.19E+05
	SRR22519794+LRG_2_75		104.495	48.052	95.110	149.246	1.418	211.620	3.99E+07	5.99E+09	1.19E+05
	SRR22519794+LRG_3_75		105.227	47.839	97.249	151.059	1.410	212.932	3.99E+07	5.99E+09	1.19E+05
	SRR22519794	1	167.240	73.646	160.135	242.974	1.416	343.968	3.32E+07	4.98E+09	1.07E+05
HRG+QLFW	QLFW	0	0.884	1.257	1.607	2.224	1.000	2.224	6.80E+07	1.02E+10	4.41E+05
	HRG+QLFW_1_25	0.226	147.035	72.580	121.432	204.041	1.523	310.666	6.59E+07	9.91E+09	3.26E+05
	HRG+QLFW_2_25		148.723	67.630	121.041	203.330	1.529	310.796	6.58E+07	9.90E+09	3.26E+05
	HRG+QLFW_3_25		146.843	71.753	121.796	203.828	1.524	310.675	6.59E+07	9.91E+09	3.25E+05
	HRG+QLFW_1_50	0.467	305.010	147.849	246.975	419.389	1.546	648.301	6.37E+07	9.58E+09	2.25E+05
	HRG+QLFW_2_50		298.888	149.015	246.582	415.141	1.543	640.662	6.37E+07	9.58E+09	2.25E+05
	HRG+QLFW_3_50		304.088	148.716	249.045	420.249	1.551	651.802	6.37E+07	9.58E+09	2.25E+05
	HRG+QLFW_1_75	0.724	474.857	233.210	384.474	653.986	1.555	1016.631	6.16E+07	9.25E+09	1.34E+05
	HRG+QLFW_2_75		476.671	231.513	382.028	653.268	1.550	1012.881	6.16E+07	9.25E+09	1.34E+05
	HRG+QLFW_3_75		466.521	232.705	386.579	649.027	1.558	1011.141	6.16E+07	9.25E+09	1.34E+05
	HRG_1_100	1	656.954	321.771	529.455	903.022	1.569	1417.003	5.95E+07	8.93E+09	2.69E+04
	HRG_2_100		660.278	321.581	528.676	904.920	1.566	1417.520	5.95E+07	8.92E+09	2.71E+04
	HRG_3_100		657.818	320.726	535.787	907.005	1.569	1423.265	5.95E+07	8.93E+09	2.70E+04

References

1. Zhao J, Guo Z, Tang L, Lu X, Li Y, Yang K. 2025. Dynamic evolution of antibiotic resistance risk in sewage sludge-amended soil during crop growth: a field-based metagenomic perspective. Environ Res 284:122799.
2. Lin Y, Zhang L, Wu J, Yang K. 2023. Wild birds-the sentinel of antibiotic resistance for urban river: Study on egrets and Jinjiang river in Chengdu, China. Environ Res 216:114566.
3. Lin Y, Dong X, Sun R, Wu J, Tian L, Rao D, Zhang L, Yang K. 2020. Migratory birds-one major source of environmental antibiotic resistance around Qinghai Lake, China. Sci Total Environ 739:139758.
4. Chng KR, Li C, Bertrand D, Ng AHQ, Kwah JS, Low HM, Tong C, Natrajan M, Zhang MH, Xu L, Ko KKK, Ho EXP, Av-Shalom TV, Teo JWP, Khor CC, Danko D, Bezdan D, Afshinnekoo E, Ahsanuddin S, Bhattacharya C, Butler DJ, Chng KR, De Filippis F, Hecht J, Kahles A, Karasikov M, Kyrpides NC, Leung MHY, Meleshko D, Mustafa H, Mutai B, Neches RY, Ng A, Nieto-Caballero M, Nikolayeva O, Nikolayeva T, Png E, Sanchez JL, Shaaban H, Sierra MA, Tong X, Young B, Alicea J, Bhattacharyya M, Blekhman R, Castro-Nallar E, Cañas AM, Chatziefthimiou AD, Crawford RW, Deng Y, et al. 2020. Cartography of opportunistic pathogens and antibiotic resistance genes in a tertiary hospital environment. Nature Medicine 26:941-951.
5. Kang Y, Wang J, Zhu C, Zheng M, Li Z. 2024. Unveiling the genomic diversity and ecological impact of phage communities in hospital wastewater. Journal of Hazardous Materials 477.
6. Roberts LW, Forde BM, Hurst T, Ling W, Nimmo GR, Bergh H, George N, Hajkowicz K, McNamara JF, Lipman J, Permana B, Schembri MA, Paterson D, Beatson SA, Harris PNA. 2021. Genomic surveillance, characterization and intervention of a polymicrobial multidrug-resistant outbreak in critical care. Microbial Genomics 7.
7. Ekwanzala MD, Dewar JB, Momba MNB. 2020. Environmental resistome risks of wastewaters and aquatic environments deciphered by shotgun metagenomic assembly. Ecotoxicology and Environmental Safety 197.
8. Elisseev V, Gardiner L-J, Krishna R. 2022. Scalable in-memory processing of omics workflows. Computational and Structural Biotechnology Journal 20:1914-1924.
9. Zhu L, Yuan L, Shuai X-Y, Lin Z-J, Sun Y-J, Zhou Z-C, Meng L-X, Ju F, Chen H. 2023. Deciphering basic and key traits of antibiotic resistome in influent and effluent of hospital wastewater treatment systems. Water Research 231.
10. Huang L, Wu X, Guo S, Lv Y, Zhou P, Huang G, Duan Z, Sun W. 2022. Metagenomic-based characterization of the gut virome in patients with polycystic ovary syndrome. Frontiers in Microbiology 13.
11. Chen L, Zhao N, Cao J, Liu X, Xu J, Ma Y, Yu Y, Zhang X, Zhang W, Guan X, Yu X, Liu Z, Fan Y, Wang Y, Liang F, Wang D, Zhao L, Song M, Wang J. 2022. Short- and long-read metagenomics expand individualized structural variations

in gut microbiomes. Nature Communications 13.
12. Zhang C, Yu L, Ma C, Jiang S, Zhang Y, Wang S, Tian F, Xue Y, Zhao J, Zhang H, Liu L, Chen W, Huang S, Zhang J, Zhai Q. 2023. A key genetic factor governing arabinan utilization in the gut microbiome alleviates constipation. Cell Host & Microbe 31:1989-2006.e8.
13. Zhang Z, Fang Y, He Y, Farag MA, Zeng M, Sun Y, Peng S, Jiang S, Zhang X, Chen K, Xu M, Han Z, Zhang J. 2024. Bifidobacterium animalis Probio-M8 improves sarcopenia physical performance by mitigating creatine restrictions imposed by microbial metabolites. npj Biofilms and Microbiomes 10.
14. Blumfelde M, Gudrā D, Začs D, Vonda K, Žorža L, Selga T, Grīnbergs A, Dēliņa A, Bartkevičs V, Fridmanis D, Muter O. 2023. Risks of Antibiotic Resistance Dissemination by Leachates from Municipal Landfills of Different Ages. Water 15.
15. Che Y, Xia Y, Liu L, Li A-D, Yang Y, Zhang T. 2019. Mobile antibiotic resistome in wastewater treatment plants revealed by Nanopore metagenomic sequencing. Microbiome 7.
16. Guajardo-Leiva S, Díez B, Rojas-Fuentes C, Chnaiderman J, Castro-Nallar E, Catril V, Ampuero M, Gaggero A. 2025. From sewage to genomes: Expanding our understanding of the urban and semi-urban wastewater RNA virome. Environmental Research 276.
17. Zhang D, Peng Y, Chan C-L, On H, Wai HK-F, Shekhawat SS, Gupta AB, Varshney AK, Chuanchuen R, Zhou X, Xia Y, Liang S, Fukuda K, Medicherla KM, Tun HM. 2021. Metagenomic Survey Reveals More Diverse and Abundant Antibiotic Resistance Genes in Municipal Wastewater Than Hospital Wastewater. Frontiers in Microbiology 12.
18. Xu R, Zhang Y, Xiong W, Sun W, Fan Q, Zhaohui Y. 2020. Metagenomic approach reveals the fate of antibiotic resistance genes in a temperature-raising anaerobic digester treating municipal sewage sludge. Journal of Cleaner Production 277.
19. Bengtsson-Palme J, Milakovic M, Švecová H, Ganjto M, Jonsson V, Grabic R, Udikovic-Kolic N. 2019. Industrial wastewater treatment plant enriches antibiotic resistance genes and alters the structure of microbial communities. Water Research 162:437-445.
20. Murphy A, Barich D, Fennessy MS, Slonczewski JL, Elliott KT. 2021. An Ohio State Scenic River Shows Elevated Antibiotic Resistance Genes, Including Acinetobacter Tetracycline and Macrolide Resistance, Downstream of Wastewater Treatment Plant Effluent. Microbiology Spectrum 9.
21. Fan X, Ji M, Mu D, Zeng X, Tian Z, Sun K, Gao R, Liu Y, He X, Wu L, Li Q. 2023. Global diversity and biogeography of DNA viral communities in activated sludge systems. Microbiome 11.
22. Sereika M, Kirkegaard RH, Karst SM, Michaelsen TY, Sørensen EA, Wollenberg RD, Albertsen M. 2021. doi:10.1101/2021.10.27.466057.
23. Zhang Q, Li J, Tuo J, Liu S, Liu Y, Liu P, Ye L, Zhang X-X. 2025. Long-term metagenomic insights into the roles of antiviral defense systems in stabilizing

activated sludge bacterial communities. The ISME Journal 19.
24. Singleton CM, Petriglieri F, Kristensen JM, Kirkegaard RH, Michaelsen TY, Andersen MH, Kondrotaite Z, Karst SM, Dueholm MS, Nielsen PH, Albertsen M. 2021. Connecting structure to function with the recovery of over 1000 high-quality metagenome-assembled genomes from activated sludge using long-read sequencing. Nature Communications 12.
25. Jouffret V, Miotello G, Culotta K, Ayrault S, Pible O, Armengaud J. 2021. Increasing the power of interpretation for soil metaproteomics data. Microbiome 9.
26. Varliero G, Anesio AM, Barker GLA. 2021. A Taxon-Wise Insight Into Rock Weathering and Nitrogen Fixation Functional Profiles of Proglacial Systems. Frontiers in Microbiology 12.
27. Fu Y, Zhang K, Shan F, Li J, Wang Y, Li X, Xu H, Qin Z, Zhang L. 2023. Metagenomic analysis of gut microbiome and resistome of Whooper and Black Swans: a one health perspective. BMC Genomics 24.
28. Peng N, Zhang J, Hu R, Liu S, Liu F, Fan Y, Yang H, Huang J, Ding J, Chen R, Li L, He Z, Wang C. 2024. Hidden pathogen risk in mature compost: Low optimal growth temperature confers pathogen survival and activity during manure composting. Journal of Hazardous Materials 480.
