# Supplementary figures and images for: MetaRanker: precise profiling of antibiotic resistome risk in metagenomes by integrating abundance and genetic co-occurrence
Source: Appl Environ Microbiol. 2026 Feb 18;92(3):e02422-25. doi: 10.1128/aem.02422-25 (PMC12997815; doi:10.1128/aem.02422-25)

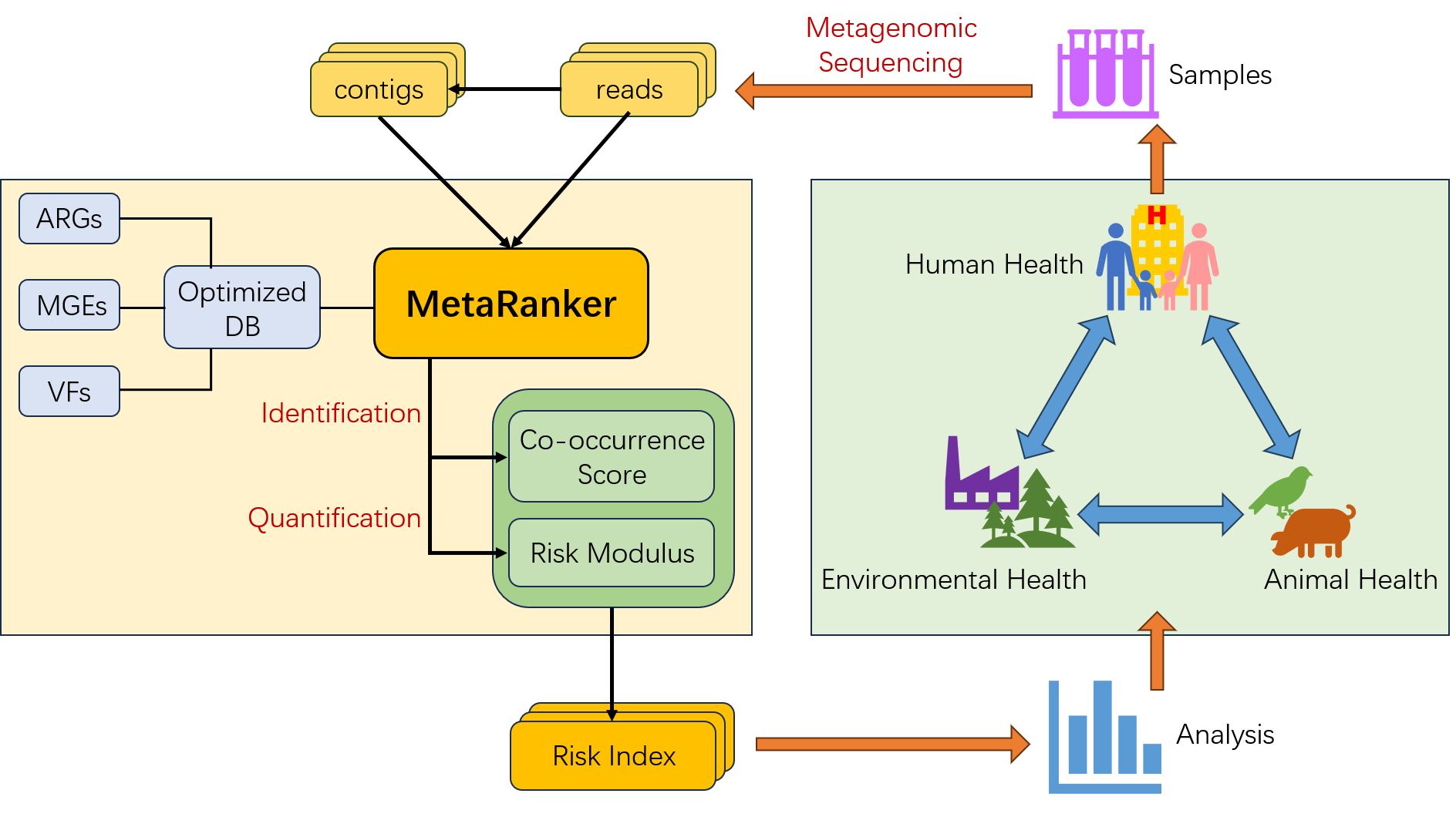

Supplement: Graphical abstract — Visual depiction of the study. [file aem.02422-25-s0002.tif]
